# Integrated metabolomic and transcriptomic analyses provide new perspectives into the discoloration of hawk tea tender leaves

**DOI:** 10.1186/s12870-026-08888-x

**Published:** 2026-05-14

**Authors:** Ximeng Yang, Hengxing Zhu, Min Lu, Feiyi Huang, Chenggong Lei, Xueping Hu, Xin Huang, Xiaolong Nie, Daojing Chen, Sicheng Huang, Benwen Chen, Qianli Dai

**Affiliations:** 1 Chongqing Academy of Forestry, Chongqing Key Laboratory of Forest Ecological Restoration and Utilization in the Three Gorges Reservoir Area, Key Laboratory of National Forestry and Grassland Administration on Phoebe Nees, Chongqing, 400036 China; 2 Observation and Research Station of Chongqing Mountain City Forest Ecosystem, Chongqing, 400036 China; 3 Wulingshan Forest Eco-Station, Chongqing, 400036 China; 4 Chongqing Zhonglinfeng Technology Development Co., Ltd, Chongqing, 400036 China

**Keywords:** Hawk tea, Tender leaves discoloration, Transcriptomics, Metabolomics, Anthocyanin

## Abstract

**Background:**

In Southwest China, the spring-colored leaves of the Lauraceae plant *Litsea coreana*, often known as hawk tea, produce a traditional beverage with cultural and economic significance*.* Its immature leaves are generally red or green coloration, transitioning to common green as they mature. The possible quality benefits of high-anthocyanin tea cultivars with unusual leaf hues have recently drawn attention. It is important from a scientific and practical standpoint to clarify the metabolism and manufacture of pigments in hawk tea leaves that have different starting hues during the developmental stages.

**Results:**

This study applied both targeted metabolomics and transcriptomics to investigate the metabolite accumulation and molecular mechanisms in red and green hawk tea tender leaves across three developmental stages. Metabolomic profiling revealed that cyanidin-3-*O*-glucoside, cyanidin-3-*O*-rutinoside, and pelargonidin-3-*O*-glucoside specifically accumulated in red tender leaves (2.44 to 4.59 Log_2_FC higher than in green leaves), with levels progressively decreasing during the red-to-green transition. Transcriptomic analysis demonstrated that key late biosynthetic genes (*ANR*, *3GT*) were consistently upregulated in red leaves and strongly correlated with anthocyanin content. In contrast, lignin pathway genes (*HCT*, *CCOAMT*) were downregulated in red leaves, suggesting that carbon flux is preferentially directed toward anthocyanin rather than lignin synthesis in red leaves. Additionally, co-expression network analysis further delineated nine candidate transcription factors (three MYB, two bHLH, three C2C2 zinc finger proteins, and one GRAS) that exhibited coordinated expression patterns with pigment biosynthetic genes, were predicted to directly or indirectly regulate anthocyanin biosynthesis in response to endogenous substances such as nitrogen, hormones, and sugars.

**Conclusion:**

Our findings identify that red leaf color in *L. coreana* is driven by anthocyanin accumulation, which is achieved through a metabolic shift that directs carbon flux away from lignin and toward anthocyanin biosynthesis. This shift is likely regulated by a set of nine transcription factors. These findings provide concrete targets for breeding high-anthocyanin hawk tea varieties.

**Supplementary Information:**

The online version contains supplementary material available at 10.1186/s12870-026-08888-x.

## Instruction

The Lauraceae family includes important economic trees that play a significant role in forestry, medicine, light industry, food, and ornamental landscaping [1]. Currently, *Cinnamomum camphora* and *C. burmannii* are the most commonly used species in landscaping, though other plant species are occasionally used with limited diversity. Many species within the Lauraceae family also have promising prospects for landscaping development largely due to their vibrant new leaf red and orange colors and their attractive tree forms, making them highly ornamental [2, 3]. Spring-colored leaf species within the Lauraceae family are primarily from genera such as *Litsea*, *Machilus*, and *Neolitsea* and are characterized by unique colors in their new shoots and tender leaves and perform well in suitable viewing environments [4–7].

Among the species in the genus *Litsea*, *Litsea coreana* var. *lanuginosa* commonly known as hawk tea is an evergreen plant whose tender leaves exhibit various colors, including red (or purple) and green just like its other relative species [8]. The tree is a unique tea raw material native to China and is primarily distributed in Chongqing, with other populations found in Sichuan and Guizhou [9, 10]. The leaves of *L. coreana* are rich in flavonoids, polyphenols, polysaccharides, and coumarins, which regulate blood sugar, protect the liver, and have anti-inflammatory, and antioxidant properties [11–13]. As a result, tea made from these natural leaves is highly favored by the local population.

The leaf color of plants results from the combined effects of various pigments, and the differences in coloration are primarily due to the varying accumulation of anthocyanins, chlorophylls, and carotenoids. Chlorophyll imparts a green color, while carotenoids contribute yellow hues, and anthocyanins provide red coloration. These colors are typically determined by the types of pigments present and their relative concentrations [14]. Anthocyanins are natural secondary metabolites soluble in water that function as antioxidants. Since they enhance the immune and have anti-inflammatory, anticancer and antitumor effects, they have received increasing attention in the fields of dietary supplements, beverages, and pharmaceuticals [15–17]. For instance, Gao et al. demonstrated the exhibition of antihypertensive, hypoglycemic, antioxidant, antiproliferative, antibacterial, and lipid metabolism-modulating effects of the high-anthocyanin Zijuang tea through pharmacological activity experiments [18]. Anthocyanins also improve vision, prevent cardio-cerebrovascular diseases, and protect the stomach [19–21]. Therefore, the red-leaf *L. coreana* is often regarded as of superior quality in local traditions and preferably produced due to its higher market price for red tender leaves.

Although research on its chemical composition [22], pharmacological effects [23], genomics [24], and ecological distribution [25] has been conducted, the substances affecting the color of *L. coreana* tender leaves and the associated biosynthetic pathways remain unclear. The application of omics technologies in the study of *L. coreana* leaf coloration has also been limited. Integrated metabolomics and transcriptomics analyses provide a systematic approach for studying the metabolites and regulatory networks in many plants. For example, Kang et al. detected a significant accumulation of lipids, especially fatty acyls and glycerophospholipids, as well as six amino acid derivatives in tea seeds, and predicted the regulatory roles of CsRAP2.10, CsWRKY2.1, CsbLHL18, and CsET2 [26]. Zhang et al. identified 47 metabolites that differ in the accumulation between yellowing and green leaves, suggesting that key genes such as *UDPG*, *HCT*, and *CsGSTF1* may play crucial roles in the process [27]. Song et al. discovered that transcription factors (TFs) like NAC008 and MYB23 may be responsible for the accumulation of flavonoids and anthocyanins in the purple tea varieties, leading to differences in leaf color compared to green leaves [28]. Zheng et al. found that the decline in chlorophyll and carotenoid content in albino tea leaves was due to changes in gene expression within the methyl erythritol 4-phosphate pathway [29]. Rothenberg et al. also used metabolomics to identify at least 12 anthocyanins in pink tea flowers and combined it with transcriptomics, revealing the molecular mechanisms underlying the rare pink flower coloration in anthocyanin-rich tea plants [30].

In this study, we combined metabolomics and transcriptomics to investigate the metabolic and regulatory networks underlying leaf color change in *L. coreana* during development. We found that three specific anthocyanins are the main pigments responsible for red color in young leaves. Transcriptomic analysis revealed that key late anthocyanin biosynthetic genes (*ANR*, *3GT*) were upregulated in red leaves, while lignin pathway genes (*HCT*, *CCOAMT*) were downregulated, indicating a shift in carbon allocation away from lignin and toward anthocyanin synthesis. This metabolic shift likely explains the higher anthocyanin accumulation in red leaves. We further identified nine transcription factors (including MYB, bHLH, and GRAS family members) that were co-expressed with these structural genes and may form a regulatory network controlling the observed metabolic shift. Our findings provide a clearer understanding of the compounds and pathways that determine leaf color in this species and offer candidate targets for future breeding efforts aimed at developing high-anthocyanin varieties with stable red leaf traits.

## Materials and methods

### Plant materials

**Fig.1:**
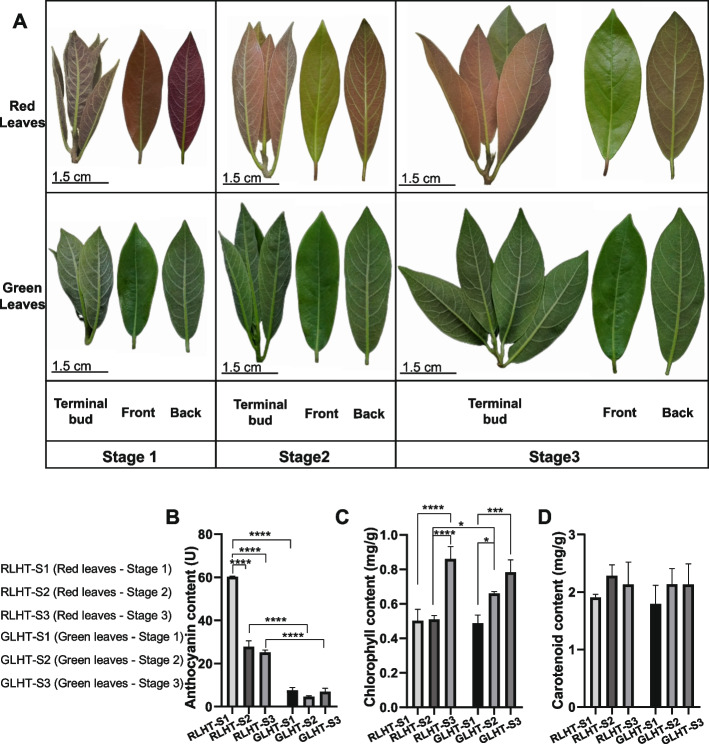
Phenotypic observations and pigment content changes in *L. coreana* leaves. **A** Morphological observations of red-leaves (R) and green-leaves (G) *L. coreana* at three developmental stages (S1-S3). Content of anthocyanins (**B**), chlorophylls (**C**), and carotenoids (**D**) in red-leaves (R) and green-leaves (G) *L. coreana* at three developmental stages (S1-S3). (*n* = 3) Values represent the mean of three independent trees, each consisting of leaves with similar coloration. Error bars represent the standard deviation of the mean. Asterisks on the bar charts indicate significant differences within and between groups, *p < 0.05, **p < 0.01, ***p < 0.001, ****p < 0.0001, analyzed by Ordinary one-way ANOVA

Fresh tender leaves were collected from six 3-year-old disease-free *L. coreana* trees grown in the nursery of the Chongqing Forestry Science Research Institute (Chongqing, China) under natural condition (20 °C, 80% relative humidity, and an 8-h photoperiod). Sampling took place from mid-March to early April 2022 (Fig. 1A, S1). The tender leaves of red phenotype (RLHT, red leaf hawk tea) and green phenotype (GLHT, green leaf hawk tea) were collected separately, and each phenotype was further subdivided into three consecutive stages based on development: Stage 1 (S1, young), Stage 2 (S2, semi-mature), and Stage 3 (S3, mature). The collected leaves were rapidly frozen in liquid nitrogen and then stored at −80℃ for subsequent analysis. All experiments were repeated, including 3 biological replicates with a total of 18 samples.

### Pigment content measurement

The contents of chlorophyll and carotenoids were extracted 0.2 g from the leaves using 95% ethanol, followed by measuring their optical density (OD) at 665, 649, and 470 nm using a UV–Vis spectrophotometer (JINGHHUA 721–100 UV–Vis spectrophotometer, Shanghai). The chlorophyll *a* content (C_Chla_) was calculated as 13.95 OD_665_−6.88 OD_649_, C_Chlb_ as 24.96 OD_649_−7.32 OD_665_, the carotenoid content (C_Car_) as (1000 OD_470_−2.05 C_Chla_−114.8 C_Chlb_)/245, and the total chlorophyll content as C_Chla_ + C_Chlb_. The pigment content was expressed as mg of pigment per gram of fresh leaf tissue (mg/g). The leaves were cut into 1–2 mm pieces, and 0.03 g was mixed with 10 mL of 0.1 mol/L hydrochloric acid (HCl) solution. Seal the flask and shake thoroughly to ensure the leaves are fully submerged in the extraction solution. Incubate in the dark for 24 h until the leaves become colorless, yielding the anthocyanin extract. The resulting extract was then analyzed using a UV spectrophotometer at wavelengths of 600 nm and 530 nm. One unit of anthocyanin is denoted as "U" and the change in OD per gram of fresh leaf extract is calculated as: OD_530_-OD_600_ = 0.1 U.

### Analysis of the anthocyanin metabolites

The anthocyanin metabolites were determined using the AB Sciex QTRAP 6500 LC–MS/MS platform (MetWare, http://www.metware.cn/). Stock solutions of 1 mg·mL^−1^ standards were prepared in 50% methanol (MeOH) and stored at −20℃. Before analysis, the stock solutions were diluted with 50% MeOH to working concentrations. Leaf samples were freeze-dried, ground at 30 Hz for 1.5 min, and stored at −80℃. Exactly 50 mg of powdered leaf sample was mixed with a solution of 0.5 mL MeOH, water and HCl prepared in the ratio of 500:500:1 (V/V/V). The mixture was vortexed and sonicated for 5 min each and then centrifuged at 12,000 g for 3 min at 4 °C. The supernatant was filtered using 0.22 µm filter paper and analysed using the ultra high performance liquid chromatography tandem mass spectrometry (UPLC-ESI–MS/MS) system (UPLC, ExionLC™ AD; MS, Applied Biosystems 6500 Triple Quadrupole). The UPLC conditions included Waters ACQUITY BEH C18 column (1.7 µm, 2.1 mm × 100 mm), solvent system consisting of water with 0.1% formic acid, methanol with 0.1% formic acid, and gradient of 95:5 at 0 min, 50:50 for 6 min, 5:95 for 12 min, hold for 2 min, 95:5 for 14 min, hold 2 min, flow rate of 0.35 mL min^−1^, temperature of 40℃, and injection volume of 2 µL. Mass spectrometry was performed using QTRAP® 6500 + LC–MS/MS with ESI Turbo Ion-Spray in positive mode. The ESI parameters included a source temperature of 550 °C, ion spray voltage of 5500 V, and curtain gas of 35 psi. Anthocyanins were analyzed using scheduled multiple reaction monitoring (MRM), with optimized transitions for each compound. Data were processed using Analyst 1.6.3 and Multiquant 3.0.3 software for quantification. Principal Component Analysis (PCA) conducted by R package factoextra (https://cloud.r-project.org/package=factoextra/) [31]. To display clusters of samples with the first two components. The differential accumulated metabolites (DAM) between different groups were determined based on the following criteria: |log2 (fold change)|≥ 2, *P* value < 0.05.

### RNA sequencing

Total RNA was extracted using a plant RNA extraction kit (Huayueyang Biotechnology Co., Ltd.). The concentration and purity were detected using NanoDrop assay (Thermo Scientific NanoDrop 2000). Total RNA with a quantity of ≥ 1 µg was selected and processed using the NEBNext Ultra II RNA Library Prep Kit for Illumina. mRNA with polyA tails was enriched using Oligo (dT) magnetic beads, followed by random fragmentation of the mRNA using divalent cations to induce ion disruption. The fragmented mRNA served as a template for cDNA synthesis, with random oligonucleotides used as primers. The resulting double-stranded cDNA was purified, then subjected to end repair and the addition of an "A" base at the 3' end, followed by ligation of sequencing adapters. cDNA fragments of approximately 400–500 bp were selected using AMPure XP beads (BECKMAN Agencourt), followed by PCR amplification. The PCR products were further purified using AMPure XP beads to obtain the final library. Library quality was assessed using the Agilent 2100 Bioanalyzer (Agilent, 2100) with the Agilent High Sensitivity DNA Kit (Agilent, 5067–4626). Samples with a RIN value ≥ 8.0 were used for subsequent library preparation (Fig. S2; Table S1). The total concentration of the library was quantified using PicoGreen (Quantiflux ST fluorometer, Promega, E6090; Quant-iT PicoGreen dsDNA Assay Kit, Invitrogen, P7589), and the effective library concentration was determined by qPCR (Thermo Scientific StepOnePlus Real-Time PCR System). Multiple DNA libraries were pooled in equal volumes, diluted, and quantified. The pooled library was sequenced in PE150 mode on an Illumina sequencer.

### RNA-Seq data analysis

The raw sequencing data in FASTQ format were filtered using fastp software (version 0.18.0) to obtain high-quality and clean reads by removing the reads containing adapters, reads with more than 10% unknown nucleotides (N), and low-quality reads with over 50% bases with a quality score (Q-value) ≤ 20 [32]. The De novo transcriptome assembly was then performed using Trinity software [33] and the gene and transcript expression levels were quantified using the RSEM software, with Transcripts Per Million (TPM) as the quantification metric. The TPM is based on the number of transcript reads, and accounts for both transcript length and the number of genes expressed in the sample. The DESeq2 [34] was used to perform differential expression analysis between multiple samples (RLHT-S2 vs RLHT-S1, RLHT-S3 vs RLHT-S2, RLHT-S1 vs GLHT-S1, RLHT-S2 vs GLHT-S2, RLHT-S3 vs GLHT-S3, GLHT-S2 vs GLHT-S1, and GLHT-S3 vs GLHT-S2). The false discovery rate (FDR) was calculated using the p-adjusted values, with thresholds set as p-adjusted < 0.05 and |log_2_ (fold change)|≥ 1 to identify differentially expressed genes (DEGs). Statistical enrichment analysis of all DEGs was conducted using the Kyoto Encyclopedia of Genes and Genomes (KEGG) database (https://www.kegg.jp/kegg/) [35].

### Protein–protein interaction (PPI) network analysis

By analyzing the domain information in gene transcription products, transcription factor prediction and family classification can be conducted. We utilized PlantTFDB 5.0 (http://planttfdb.gao-lab.org/) to perform transcription factor analysis on genes derived from plants. The PPI network analysis of the genes of interest was performed using the STRING database (http://string-db.org/) [36]. We used the well-established model organism *Arabidopsis thaliana* with comprehensive protein interaction data and directly extracted the interaction relationships corresponding to the genes of interest from the database to construct the PPI network. Subsequently, the Pearson correlation and Euclidean distance algorithms were applied to calculate the Pearson correlation coefficients (R^2^) and p-values by integrating transcriptome TPM data with the anthocyanin metabolome data. A correlation coefficient > 0.7 was selected to construct a visual co-expression network, which was visualized using Cytoscape software version 2.8 [36].

### qRT-PCR analysis

The qRT-PCR experiments were performed in triplicates according to the instructions of the TB Green Premix Ex Taq II (Tli RNaseH Plus) kit on a Bio-Rad iCycler thermal cycler (Bio-Rad, Hercules, CA, USA). The qPCR primers (Table S2) for *L. coreana* genes were designed using Primer 5.0 software (Premier Biosoft International, Palo Alto, California, US). The *actin* gene was selected as the internal reference gene [37]. The melting curve exhibited a single peak (Fig. S3), indicating that specific amplification products were melting at a specific temperature. Relative gene expression levels were calculated using the 2^−ΔΔCT^.

### Statistical analyses

Line charts, bar graphs, and heat maps were generated using GraphPad 8.0. Statistical significance was assessed using one-way ANOVA with SPSS 25 software (IBM, Chicago, IL, USA). A *p*-value less than 0.05 was considered statistically significant.

## Results

### Leaf color changes at different developmental stages

This study determined the pigment contents in leaves of red-leaf Hawk Tea (RLHT) and green-leaf Hawk Tea (GLHT) across three developmental stages (S1, S2, and S3) (Fig. 1A). S1 (Young stage), leaves with leaf margins inwardly folded or revolute and not yet fully flattened. The leaf blade is soft and tender, with obvious trichomes on the abaxial surface; S2 (Semi-mature stage), leaves are fully flattened, the leaf texture remains relatively soft and the glossiness of the leaf surface increases; S3 (Mature stage), leaves have reached maximum leaf area, with a hard and brittle texture, and an thickened waxy cuticle on the leaf surface.

The anthocyanin content was significantly higher in RLHT than in GLHT at all three stages (*p* < 0.05). In RLHT, the anthocyanin level was highest at S1, reaching 60.36 U/g FW, then gradually declined to 27.86 U/g FW at S2 and further to 25.22 U/g FW at S3, mirroring the visible color shift from red to green. In contrast, GLHT maintained consistently low anthocyanin levels throughout development, with an average of 6.44 U/g FW (Fig. 1B).

Chlorophyll content increased progressively from S1 to S3 in both lines, a pattern that reflects leaf maturation and the gradual establishment of photosynthetic capacity. Notably, despite their red appearance at early stages, RLHT leaves showed no significant difference in chlorophyll content from GLHT leaves at the corresponding stages. For instance, at S1, RLHT and GLHT contained 0.50 mg/g and 0.49 mg/g of chlorophyll, respectively (*p* > 0.05), indicating that active chlorophyll synthesis still occurred in the red leaves. However, by S3, RLHT exhibited significantly higher chlorophyll content than S1, with values of 0.86 mg/g (*p* < 0.05) (Fig. 1C).

Carotenoid content remained largely stable across stages and between the two lines, with only minor fluctuations. At S1 and S2, no significant differences were detected between RLHT and GLHT. At S2, both RLHT and GLHT showed a slight increase in carotenoid levels compared to S1 but the difference was not statistically significant (RLHT-S1: 1.90 mg/g, RLHT-S2: 2.28 mg/g; *p* > 0.05). Overall, no consistent directional trend was observed for carotenoid content across development (Fig. 1D).

### Identification of anthocyanin metabolites in L. coreana leaves

**Fig. 2:**
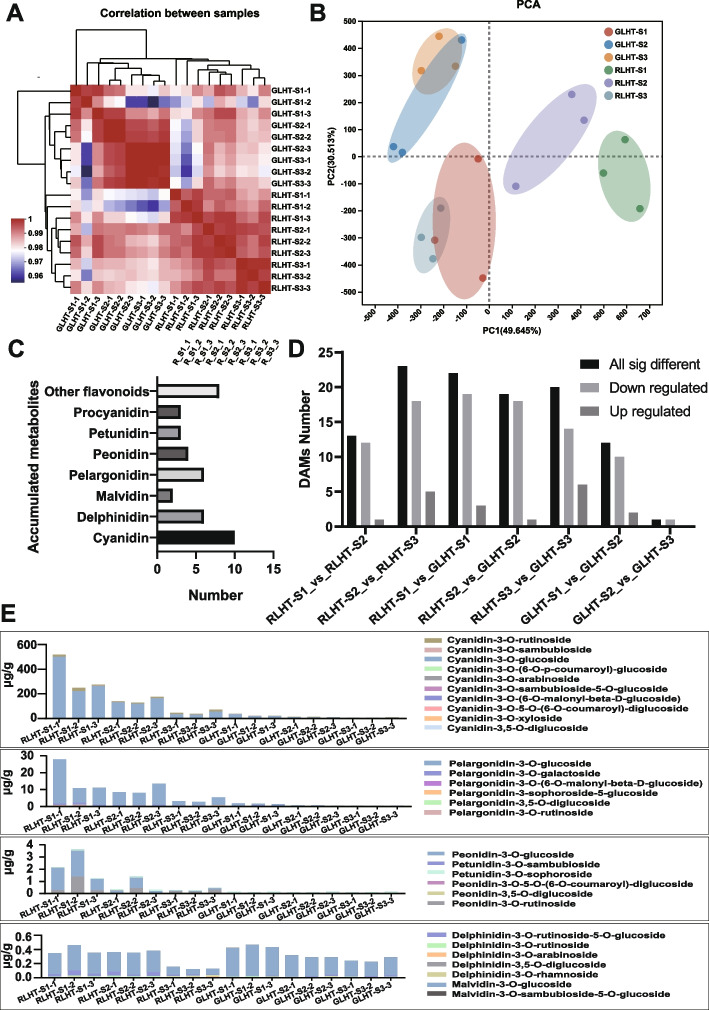
Metabolomic Analysis of tender leaves in *L. coreana*. **A** Sample correlation analysis. The closer the correlation coefficient is to 1, the stronger the correlation. **B** Principal component analysis of metabolomic data from leaves of different colored *L. coreana*. **C** Classification of metabolites in the leaves. The x-axis represents the quantity of each flavonoid metabolite. **D** Differentially accumulated metabolites (DAMs) in the seven comparisons: RLHT-S1 vs RLHT-S2, RLHT-S2 vs RLHT-S3, RLHT-S1 vs GLHT-S1, RLHT-S2 vs GLHT-S2, RLHT-S3 vs GLHT-S3, GLHT-S1 vs GLHT-S2, and GLHT-S2 vs GLHT-S3. **E** Analysis of the absolute content of significantly differentially accumulated anthocyanin metabolites

The corresponding heatmap demonstrated a strong correlation between the three biological replicates used for each sample (Fig. 2A). The principal component analysis (PCA) revealed a clear separation between red and green leaves along the first principal component (PC1), which explained 49.65% of the variation (Fig. 2B). The distinct separation between red and green leaves in the PCA indicates significant differences in metabolite profiles between leaf color variants, as well as across the three developmental stages of the leaves. All the analyses confirmed the reliability of the metabolomic data.

A total of 42 metabolites were identified, including 31 anthocyanins, 3 proanthocyanidins and 8 other flavonoids (Fig. 2C; Table S3). Differentially accumulated metabolites (DAMs) were analyzed in pairwise comparisons across seven groups including RLHT-S1 vs RLHT-S2, RLHT-S2 vs RLHT-S3, RLHT-S1 vs GLHT-S1, RLHT-S2 vs GLHT-S2, RLHT-S3 vs GLHT-S3, GLHT-S1 vs GLHT-S2, and GLHT-S2 vs GLHT-S3. Thirteen, 23, 22, 19, 20, 12, and 1 DAMs were identified in each respective comparison, with a total of 29 DAMs across the groups (Fig. 2D; Table S4). The analysis of the absolute content of significantly accumulated anthocyanins in leaves with different developmental stages and leaf colors revealed that cyanidin-3-*O*-glucoside (C3G), cyanidin-3-*O*-rutinoside (C3R) and pelargonidin-3-*O*-glucoside (P3G) were the predominant anthocyanins in the red *L. coreana* tender leaves. The contents of these anthocyanins were significantly higher in red than green tender leaves (Table S3) but gradually decreased as the leaf developed (Fig. 2E). The expression patterns of these anthocyanin metabolites were also consistent with the phenotypic changes, suggesting that C3G, C3R and P3G may be key metabolites associated with the formation of red tender leaves in *L. coreana*.

### Transcriptome data quality and DEGs analysis

To further investigate the color development mechanism in *L. coreana* leaves, transcriptomic analysis was performed. A total of 18 samples yielded 121.05 GB of clean data, with each sample generating more than 5.76 GB, with a Q30 base percentage exceeding 93.01% (Table S5). Further filtering of sequencing data, data filtering Clean Data range from 92.90 to 93.38% (Table S6).

**Fig. 3:**
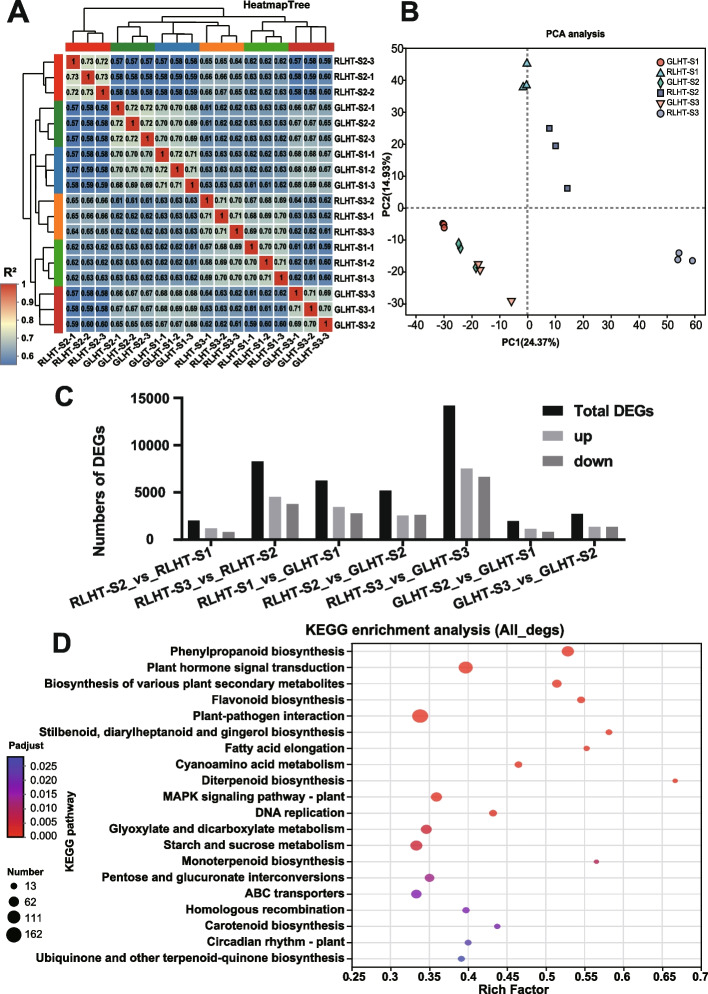
Transcriptomic analysis of *L. coreana* tender leaves. **A** Sample correlation heatmap based on transcriptomic data from 18 samples with different leaf colors and developmental stages. **B** Principal component analysis (PCA) of transcriptomic data from 18 samples. **C** Differentially expressed genes (DEGs) statistics for different comparisons. **D** KEGG enrichment pathways for all DEGs in the various comparisons

Sample correlation analysis based on RNA-seq data yielded Spearman correlation coefficients ranging from approximately 0.65 to 0.75 among biological replicates (Fig. 3A). While this indicates a moderate degree of inherent biological and technical variation, unsupervised hierarchical clustering clearly grouped all biological replicates within their respective phenotype and developmental stage groups.

Additionally, the PCA revealed clustering within groups and clear separation between different groups, with PC1 accounting for 24.37%, primarily distinguishing green from red tender leaves and PC2 accounting for 14.93%, with a strong separation between visually distinct red and green leaves (Fig. 3B). While the cumulative variance (39.3%) may appear modest in absolute terms, it clearly separates the two core biological factors of interest. Notably, RLHT S3 was positioned separately from RLHT S1 and S2 in the PCA plot, falling in the fourth quadrant while the earlier stages clustered in the first quadrant, with all GLHT samples grouped in the third quadrant. This separation likely reflects the advanced maturation of RLHT S3, during which anthocyanin declines and the transcriptional program shifts toward a “mature leaf” state more similar to green leaves than to its own earlier stages, consistent with our pigment data. Thus, these PCA results suggest high reproducibility of the data within groups and significant variation between groups.

A total of 21,445 DEGs were identified using DESeq2. Among these, 2,004 DEGs were detected in the comparison between the S1 and S2 developmental stages of RLHT, with 1,211 genes up-regulated and 793 down-regulated. A total of 8,285 DEGs were identified between the S2 and S3 developmental stages of RLHT, with 4,519 genes up-regulated and 3,766 down-regulated. A comparison of the first developmental stage of RLHT with the S1 developmental stage of GLHT revealed 6,252 DEGs, with 3,462 genes up-regulated and 2,790 down-regulated. A comparison of the S2 developmental stage of RLHT with the second developmental stage of GLHT, identified 5,181 DEGs, with 2,558 genes up-regulated and 2,623 down-regulated. The comparison between the S3 developmental stage of RLHT and the third developmental stage of GLHT revealed 14,167 DEGs, with 7,519 genes up-regulated and 6,648 down-regulated. The comparison of the S1 and S2 developmental stages of GLHT showed 1,967 DEGs, with 1,144 genes up-regulated and 829 down-regulated, while the comparison between the S2 and S3 developmental stages of GLHT revealed 2,732 DEGs, with 1,370 genes up-regulated and 1,362 down-regulated (Fig. 3C).

### Analysis of key structural genes associated with the accumulation of three pigment types

To explore the molecular basis of pigment accumulation in red and green hawk tea leaves, we performed KEGG pathway enrichment analysis on the differentially expressed genes (DEGs) identified between RLHT and GLHT across the three developmental stages.

As shown in Fig. 3D and Table S7, the most strongly enriched pathways were phenylpropanoid biosynthesis (ko00940, 93 DEGs, *P* = 2.00 × 10^–16^) and flavonoid biosynthesis (ko00941, 36 DEGs, *P* = 1.11 × 10^–7^), both of which are upstream of anthocyanin synthesis. Anthocyanin biosynthesis (ko00942) itself was also significantly enriched (*P* = 0.046), though with only three DEGs, reflecting its narrow and terminal nature.

For chlorophyll-related metabolism, porphyrin metabolism (ko00860) was not statistically significant after FDR correction (*P*-adjust = 0.27), but we still observed 28 DEGs involved in chlorophyll biosynthesis, the chlorophyll cycle, and chlorophyll degradation (Fig. S4B). This is consistent with the pigment data showing clear changes in chlorophyll content during leaf development, even though the overall pathway enrichment did not reach the significance threshold.

Carotenoid biosynthesis (ko00906) was significantly enriched (*P* = 0.0023, *P*-adjust = 0.019), with 21 DEGs encoding 10 enzymes (Fig. S4C). Its enrichment level was lower than that of the flavonoid pathways, matching the pigment data in Fig. 1D, where carotenoid levels showed only minor variation across stages and between the two color types.

A closer examination of the DEGs within these pathways revealed a comprehensive picture of the molecular machinery involved. In the anthocyanin biosynthesis pathway, we identified 39 DEGs encoding 12 structural enzymes (Fig. S4A), including 2 *C4H*, 7 *CHS*, 1 *F3H*, 1 *F3'H*, 2 *ANS*, 1 *FLS*, 2 *LAR*, 1 *ANR*, 2 *C3'H*, 4 *CCOAMT*, 13 *HCT*, 3 *3-GT* genes. Additionally, we identified 28 genes encoding 23 enzymes involved in three chlorophyll metabolic pathways (Fig. S4B), including chlorophyll biosynthesis, chlorophyll cycle and chlorophyll degradation pathways. In the chlorophyll biosynthesis pathway, 1 *HEMA*, 1 *HEML*, 1 *HEMC*, 2 *HEMD*, 2 *HEME*, 1 *HEMF*, 2 *HEMH*, 1 *CHLH*, 1 *CHLE*, 1 *CHLI*, 1 *CHLP*, and 1 *POR* genes were found. In the chlorophyll cycle pathway, we identified 1 *CHLG*, 1 *HCAR*, 2 *NOL*, 1 *CAO*, and 2 *CLH*, while in the chlorophyll degradation pathway, 1 *EARS*, 1 *cobA*, 1 *HO*, 1 *RCCR*, 1 *SGR*, and 1 *UGT* gene were identified. We also identified 21 DEGs encoding 10 enzymes related to carotenoid biosynthesis (Fig. S4C), including 3 *crtB*, 2 *CCD7*, 1 *CYP97C1*, 1 *crtZ*, 3 *ABA1*, 1 *CCS1*, 3 *NCED*,1 *ABA2*, 1 *AAO*, and 5 *CYP707A* genes (Table 1).

**Table 1:** Leaf color-related metabolic pathways in *L. coreana*

Number	Pathway	Gene count	Pathway ID
1	Flavonoid biosynthesis	36	ko00941
2	Anthocyanin biosynthesis	3	ko00942
3	Porphyrin and chlorophyll metabolism	28	ko00860
4	Carotenoid biosynthesis	21	ko00906

### Identification of transcription factors involved in pigment synthesis metabolism

**Fig. 4:**
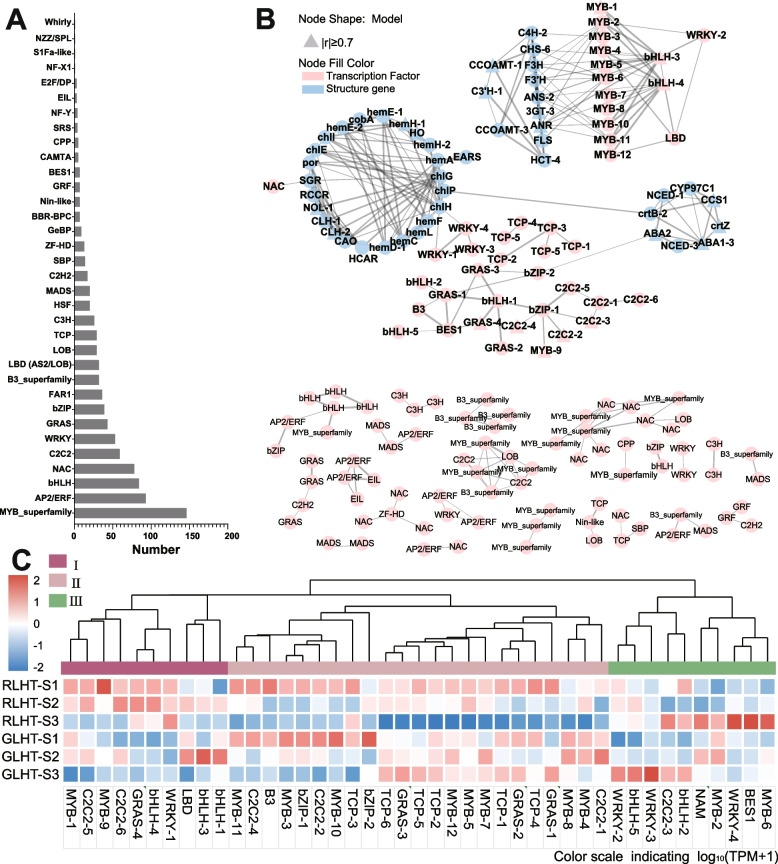
Transcription factors involved in the synthesis of three pigment types. **A** The number of genes of different transcription factor types within the differential genes. **B** Protein–protein interaction (PPI) network analysis between 88 pigment-related genes and 928 transcription factor-encoding genes. The thickness of the connecting lines represents the combined score of interactions between gene-encoded proteins, with thicker lines indicating stronger interactions. Genes marked with triangles are significantly correlated with pigment content (|r|> 0.7). **C** Heatmap of the differential expression patterns of the 42 transcription factors, after excluding those that did not form clusters with structural genes

The transcription factor (TF) analysis performed on the assembled genes identified 928 TFs belonging to 21 TF families (Fig. 4A), with v-myb myeloblastosis viral oncogene homolog (MYB), APETALA2/ethylene-responsive element binding factor (AP2), and basic helix-loop-helix (bHLH) being the top three most abundant TF families. To precisely select TFs involved in the synthesis of the three pigment types, we performed a PPI network analysis of 88 genes, including the 39 genes related to anthocyanin synthesis, 28 genes related to chlorophyll metabolism, and 21 genes involved in carotenoid synthesis with the 928 TFs (Fig. 4B). Using *A. thaliana* as a reference species, we selected interactions with a combined score greater than or equal to 0.4 and the top 300 interacting pairs for Cytoscape visualization. After excluding those that did not form clusters with structural genes, we identified 43 TFs (Fig. 4C), including 12 that encode MYB-type TFs and five that encode bHLH-type TFs. Using Euclidean distance-based hierarchical clustering of gene expression profiles, we found that 43 transcription factors (TFs) were grouped into three clusters. Cluster I exhibited prominent expression specifically during the S1 and S2 in RLHT. Cluster II showed prominent expression during S1 and S2 in both RLHT and GLHT. Cluster III, however, was characterized by prominent expression either in RLHT or specifically during the S3 in GLHT.

### Correlation analysis between metabolomics and transcriptomics

**Fig. 5:**
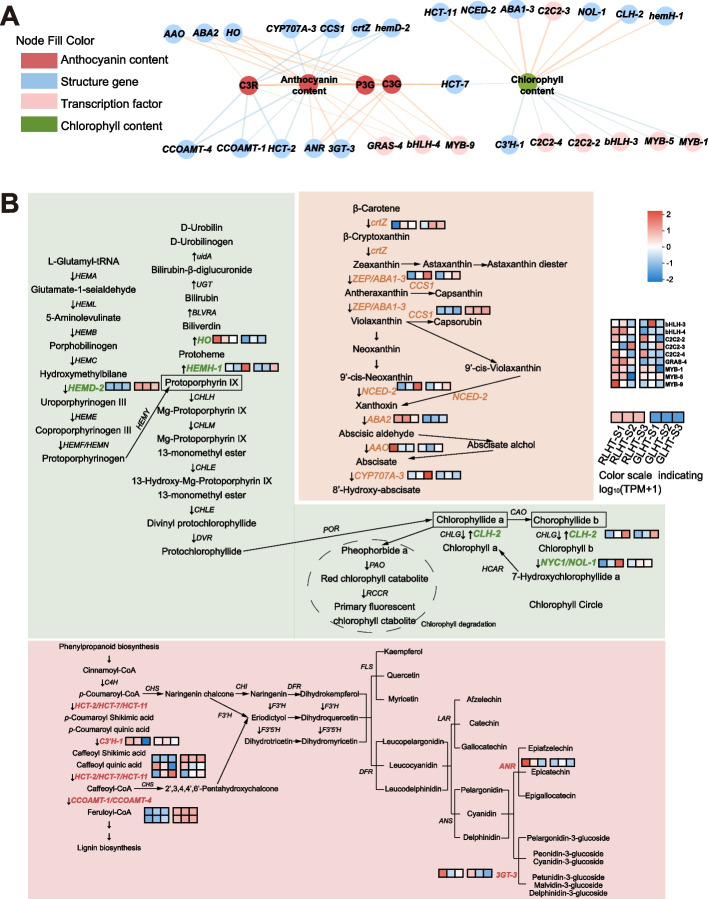
Integrated map of pigment metabolism pathway and correlation network. **A** Correlation network of pigment synthesis-related genes and metabolites in *L. coreana* Leaves. The thickness of the lines represents the strength of the correlation coefficient, with thicker lines indicating higher absolute values. The line color represents the type of correlation: red lines indicate positive correlations, and blue lines indicate negative correlations. **B** Heatmap of differentially expressed genes significantly correlated with pigment content. The green box highlights the Porphyrin and Chlorophyll Metabolism pathway, the orange box highlights the Carotenoid Biosynthesis pathway, the red box highlights the Anthocyanin Biosynthesis pathway

To further identify genes involved in pigment synthesis in *L. coreana* leaves, we conducted a correlation network analysis by evaluating the Spearman correlations among DEGs, DAMs, and pigment content (Table S8). A threshold of *p* < 0.05 and |r|> 0.7 was used to filter the network (Fig. 5A, Table 2). Based on this, we identified six structural genes (*ANR*, *3GT-3*, *HCT-7*, *HO*, *AAO*, *ABA2*) and seven (*CCOAMT-1*, *CCOAMT-4*, *HCT-2*, *hemD-2*, *CCS1*, *CYP707A-3*, *crtZ*) anthocyanin metabolites that positively and negatively correlated with anthocyanin content, respectively. Additionally, three TFs (*MYB-9*, *bHLH-4*, *GRAS-4*) were positively correlated with anthocyanin content (Fig. 5A). Similarly, six structural genes (*CLH-2*, *hemH-1*, *NOL-1*, *NCED-2*, *ABA1-3*, *HCT-11*) and one TF (*C2C2-3*)were positively correlated with chlorophyll content, while two structural genes (*C3’H-1*, *HCT-7*) and five TFs (*MYB-1*, *MYB-5*, *bHLH-3*, *C2C2-2*, *C2C2-4*) were negatively correlated with chlorophyll content (Fig. 5A). No genes meeting the correlation criteria were identified for carotenoid content. However, the contents of the total anthocyanin, C3G and P3G showed significant positive correlations with the *3GT-3* gene involved in anthocyanin biosynthesis, *HCT-7* gene in the flavonoid biosynthesis pathway, and the TFs *MYB-9* and *bHLH-1*. Interestingly, *HCT-7* was also significantly negatively correlated with chlorophyll content, and the TFs *MYB-1*, *MYB-5*, and *bHLH-1*. Interestingly, in the hierarchical clustering analysis, the TFs MYB-1, MYB-5, and bHLH-1 were also classified into the same cluster (Fig, 4 C).

**Table 2:** Key differentially expressed genes, transcription factors, and metabolites involved in pigment metabolism in *L. coreana*

Annotation	Relative for Anthocyanin	Relative for Chlorophyll	Pathway id	Pathway definition
*hemD-2*	negative	none	map00860; map01240	Porphyrin metabolism; Biosynthesis of cofactors
*hemH-1*	none	positive	map00860; map01240	Porphyrin metabolism; Biosynthesis of cofactors
*NOL-1*	none	positive	map00860	Porphyrin metabolism
*CLH-2*	none	positive	map00860	Porphyrin metabolism
*HO*	positive	none	map00860	Porphyrin metabolism
*AAO*	positive	none	map00906; map00380	Carotenoid biosynthesis; Tryptophan metabolism
*ABA2*	negative	none	map00906	Carotenoid biosynthesis
*CCS1*	negative	none	map00906	Carotenoid biosynthesis
*crtZ*	negative	none	map00906	Carotenoid biosynthesis
*CYP707A-3*	negative	none	map00906	Carotenoid biosynthesis
*NCED-2*	none	positive	map00906	Carotenoid biosynthesis
*ABA1-3*	none	positive	map00906	Carotenoid biosynthesis
*3GT-3*	positive	none	map00942	Anthocyanin biosynthesis
*ANR*	positive	none	map00941	Flavonoid biosynthesis
*C3'H-1*	none	negative	map00941; map00940; map00945	Flavonoid biosynthesis; Phenylpropanoid biosynthesis; Stilbenoid, diarylheptanoid and gingerol biosynthesis
*CCOAMT-1*	negative	none	map00941; map00940; map00945	Flavonoid biosynthesis; Phenylpropanoid biosynthesis; Stilbenoid, diarylheptanoid and gingerol biosynthesis
*CCOAMT-4*	negative	none	map00941; map00940; map00945	Flavonoid biosynthesis; Phenylpropanoid biosynthesis; Stilbenoid, diarylheptanoid and gingerol biosynthesis
*HCT-2*	negative	none	map00941; map00940; map00945	Flavonoid biosynthesis; Phenylpropanoid biosynthesis; Stilbenoid, diarylheptanoid and gingerol biosynthesis
*HCT-7*	positive	negative	map00941; map00940; map00945	Flavonoid biosynthesis; Phenylpropanoid biosynthesis; Stilbenoid, diarylheptanoid and gingerol biosynthesis
*HCT-11*	none	positive	map00941; map00940; map00945	Flavonoid biosynthesis; Phenylpropanoid biosynthesis; Stilbenoid, diarylheptanoid and gingerol biosynthesis
*bHLH-3*	none	negative	bHLH	-
*bHLH-4*	positive	none	bHLH	-
*C2C2-2*	none	negative	C2C2	-
*C2C2-3*	none	positive	C2C2	-
*C2C2-4*	none	negative	C2C2	-
*GRAS-4*	positive	none	GRAS	-
*MYB-1*	negative	none	MYB_superfamily	-
*MYB-5*	negative	none	MYB_superfamily	-
*MYB-9*	positive	none	MYB_superfamily	-

Simultaneously, analysis of the expression profiles of structural genes revealed a cross-pathway transcriptional coordination network in the red and green leaf types (Fig. 5B). In the chlorophyll synthesis pathway, the expression of the gene *hemD-2* showed a significant negative correlation with anthocyanin content, and its expression level was significantly lower in all three developmental stages of RLHT compared to the corresponding stages in GLHT. Conversely, the expression of the *HO* gene exhibited a significant positive correlation with anthocyanin content and remained consistently highly expressed across the three stages in RLHT. A similar divergent pattern was observed in the carotenoid/abscisic acid (ABA) biosynthesis pathway: the expression of *CCS1* correlated negatively with anthocyanin and was lower in RLHT across all stages, whereas *ABA2*, a gene involved in ABA synthesis, correlated positively with anthocyanin and showed higher expression in RLHT. Regarding the upstream competition for precursors leading to anthocyanins, the expression of key genes in the lignin branch (*HCT-2*, *CCOAMT-1*, and *CCOAMT-4*) all showed significant negative correlations with anthocyanin content, and their expression was comprehensively lower in red leaves than in green leaves. This strongly suggests that during red leaf development, carbon flux is preferentially directed towards anthocyanin rather than lignin biosynthesis, forming a clear metabolic diversion.

To understand the specific positions of these genes within their respective pigment synthesis pathways, we examined the expression of biosynthetic pathway genes. During leaf development, limited precursors in green tissue appear to preferentially direct flux toward lignin biosynthesis.

### qRT-PCR verification of RNA-Seq analysis

**Fig. 6:**
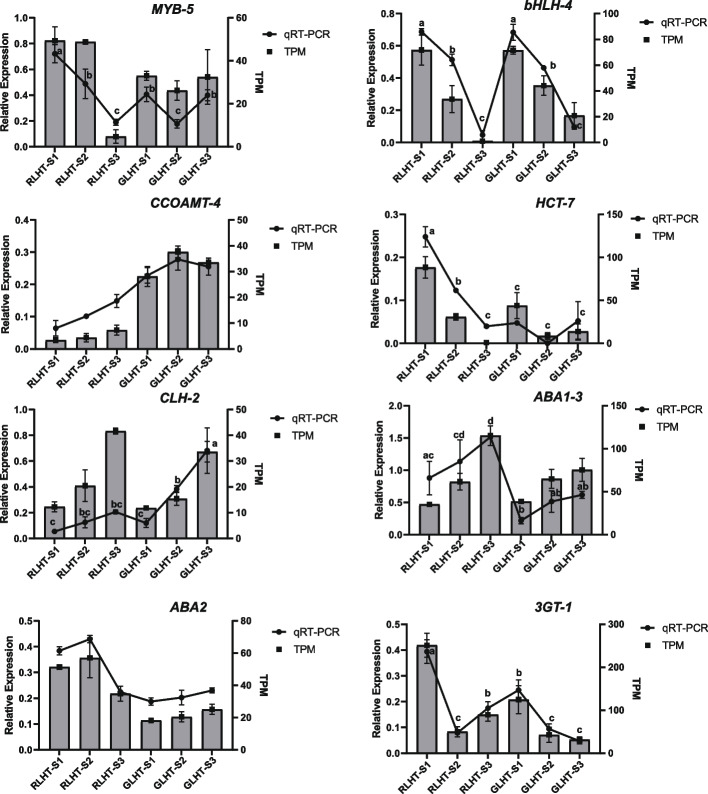
qRT-PCR validation of RNA-Seq data. The right y-axis represents the TPM values obtained from RNA-Seq. The left y-axis shows the relative gene expression levels measured by qRT-PCR. Error bars represent the standard error of the mean (SE) (*n* = 3). Different letters above the bars indicate statistically significant differences in relative gene expression levels measured by qRT-PCR (*p* < 0.05)

We selected eight key genes from the correlation analysis to validate the transcriptomics data using the qRT-PCR. The results showed that the qRT-PCR data were consistent with the TPM values, confirming the reliability of the transcriptomic data (Fig. 6). We also observed significantly higher levels of *MYB-5* and *HCT-7* genes in RLHT compared to GLHT at all developmental stages, further supporting the involvement of *MYB-5* and *HCT-7* as key factors in the pigment formation in *L. coreana* leaves.

## Discussion

### Anthocyanin content as the key factor determining leaf color in L. coreana

Chlorophyll, carotenoids, and anthocyanins are the pigments that primarily influence leaf color in plants. In our study, we measured these pigments across three developmental stages in red-leaf (RLHT) and green-leaf (GLHT) hawk tea (Fig. 1). In RLHT, anthocyanin content was high at the early stage and gradually decreased as leaves matured, closely matching the visible color shift from red to green. In contrast, GLHT maintained consistently low anthocyanin levels throughout development. Chlorophyll content increased progressively in both lines from S1 to S3, reflecting leaf maturation and the establishment of photosynthetic capacity. Notably, although RLHT leaves appeared red at early stages, their chlorophyll content did not differ significantly from GLHT at the same stages, indicating that chlorophyll synthesis was still active in red leaves. Carotenoid levels showed little variation across stages and between the two lines.

These patterns indicate that the red coloration of RLHT young leaves is primarily driven by anthocyanin accumulation rather than by differences in chlorophyll or carotenoid content. Similar findings have been reported in other plants. For instance, in *Alternanthera bettzickiana*, the green-leaved varieties are characterized by low anthocyanin content, with mature chloroplasts giving the leaves a green color, while the red varieties have low chlorophyll content, deformed chloroplasts, and higher anthocyanin content, resulting in red leaved plants [38]. In the *Anthurium andraeanum*, the formation of leaf color is largely influenced by chloroplast development and pigment biosynthesis activity [39], while in *Brassica juncea*, the purple leaf phenotype is due to an increase in anthocyanins and suppression of chlorophyll synthesis [40]. In *Triadica sebifera*, green-leafed plants primarily contain chlorophyll and carotenoids, which decrease during the purple-leaf phase as the anthocyanin levels increase, enhancing the purple appearance of the leaves [41]. Therefore, compared to these plants, *L. coreana* exhibits a distinct leaf coloration pattern in which the final red color of its tender leaves is less related to chlorophyll and carotenoids but is primarily driven by the anthocyanin content.

Thus, our findings provide further evidence that leaf color is one of the most desirable traits in urban trees that is determined by anthocyanins [14]. Over 650 distinct anthocyanins have been identified in nature, with the most common being cyanidin, delphinidin, petunidin, peonidin, malvidin, and pelargonidin. Among the anthocyanins, cyanidin and pelargonidin are associated with a red or deep red coloration [17, 42]. Consistent with these findings, we identified two cyanidins C3G and C3R, and a pelargonidin P3G as the predominant anthocyanins with significantly higher concentrations in RLHT than in GLHT, indicating that these anthocyanins play key roles in accumulating red pigments in the leaves of *L. coreana*.

### The differences and similarities between red and green tender leaves

The role of anthocyanin biosynthesis in leaf development has been extensively studied across various plant species, including monocotyledonous ornamental plant *Caladium* [43, 44], vegetable *Brassica* [40, 45], tea tree *Camellia sinensis* [28], as well as autumn leaf tree species such as *Acer pictum* subsp. *mono* [46], *A. palmatum* [47], and *Liquidambar Formosana* [48]. In our current study, several key structural genes involved in late anthocyanin biosynthetic genes such as *ANR* and *3GT*, were differentially expressed during leaf development (Fig. 5). The expression of *3GT* and *ANR* genes strongly correlated with anthocyanin content in young red leaves of *L. coreana*, suggesting their critical role in accumulating anthocyanin. The two genes share the same substrate, producing colored anthocyanins using anthocyanidin synthase. The produced anthocyanins are unstable without glycosylation, potentially forming proanthocyanidins via *ANR* or stable anthocyanins via *3GT*.

Lignin and anthocyanin share common precursors, which may explain the color differences between red and green leaves. *HCT* and *CCOAMT* are key enzymes in the lignin biosynthesis pathway [49, 50]. They compete with *CHS* (chalcone synthase), the first key enzyme in the anthocyanin biosynthesis pathway, for common upstream substrates such as coumaroyl-CoA and caffeoyl-CoA. In RLHT, the expression levels of these genes were significantly lower than in GLHT, indicating a reduced carbon flux towards lignin biosynthesis (Fig. 5). This suppression allows more precursors to enter the flavonoid pathway, providing raw material for anthocyanin synthesis. This represents a classic mechanism of metabolic channeling. Similar regulatory patterns have been reported in other plants. For instance, in *Populus*, overexpression of miR156 or MYB6 promotes flavonoid and anthocyanin accumulation while suppressing lignin biosynthesis [51, 52]. Conversely, the overexpression of *ZmMYB42* increased H- and G-type lignin content but decreased the S-type lignin, flavonols, and anthocyanin [53, 54]. These findings highlight the regulation of the competitive relationship between lignin and anthocyanin biosynthesis in plants by upstream TFs and epigenetic modifications.

In addition to the anthocyanin and lignin pathways, we also looked at genes involved in chlorophyll and carotenoid metabolism. KEGG enrichment showed that “porphyrin metabolism” (ko00860), which covers chlorophyll biosynthesis and degradation, did not reach statistical significance after FDR correction (*p*-adjust = 0.27) (Table S7, Fig. 3D). Among them, *HEMD* was downregulated in RLHT, while *HO* was upregulated and its expression tracked with anthocyanin content. For carotenoid biosynthesis (ko00906), 21 DEGs were significantly enriched (*p*-adjust = 0.019), and *ABA2* showed higher expression in RLHT, also correlating with anthocyanin (Fig. S4C). The weaker enrichment of these pathways compared to flavonoid biosynthesis fits with the pigment data (Fig. 1), where chlorophyll and carotenoid levels showed little difference between red and green leaves at early stages, suggesting they play a supporting rather than a primary role in the red-green color difference.

### The fading of red color in L. coreana leaves

The downregulation of anthocyanin biosynthesis-related TFs and structural genes may contribute to the fading of red color in *L. coreana* leaves. For example, in our study, the expression levels of structural genes involved in anthocyanin biosynthesis, including *C4H-1*, *CHS-1* to *CHS-7*, *F3'H*, *F3H*, *ANS-1*, and *ANS-2*, gradually decreased during leaf development and could have led to the reduced accumulation of anthocyanins, resulting in the gradual fading of the leaf color.

Anthocyanin biosynthesis is regulated by the MBW complex, which consists of R2R3-MYB, bHLH, and WD40 transcription factors [15–17]. The MBW complex consists of R2R3-MYB, bHLH, and WD40 TFs, but its function is primarily controlled by the activity of R2R3-MYB genes, which promote or inhibit the transcription of structural genes [41, 52]. In our study, three R2R3-MYB genes (*MYB-1*, *MYB-5*, and *MYB-9*) showed decreasing expression from S1 to S3 in RLHT (Fig. 5C). Among them, *MYB-1* and *MYB-5* also showed relatively high expression in green leaves and were negatively correlated with chlorophyll content. This suggests that they may require bHLH partners, such as *bHLH-4* identified in this study, to effectively regulate anthocyanin accumulation.

We also observed that *HO* expression paralleled anthocyanin content (Fig. 5). *HO* catalyzes heme degradation and is not directly involved in anthocyanin synthesis. Its role in leaf color formation may be interpreted in two ways. First, in RLHT, *HEMD* expression is suppressed (Fig. S4B), likely reducing flux through the tetrapyrrole pathway and leading to heme accumulation. Highly expressed *HO* may accelerate heme breakdown, generating signaling molecules such as biliverdin, CO, and Fe^2+^, which can activate MYB and bHLH transcription factors to promote anthocyanin synthesis [55]. Thus, high HO expression in early-stage RLHT may act as a signal amplifier. Second, a common upstream transcription factor, such as a HY5-like factor homologous to *bZIP-1* in this study, may regulate both *HO* and anthocyanin pathway genes [55, 56]. This is consistent with our observation of the coordinated decline in *bZIP-1* expression, *HO* expression, and anthocyanin content during RLHT development, which gradually weakened as the RLHT underwent developmental fading.

### Endogenous factors influencing anthocyanin accumulation and leaf coloration in L. coreana

Though anthocyanin is the main pigment that influences the leaf color of *L. coreana* tender leaves, a combined transcriptomic and metabolomic analysis revealed a weak correlation between the expression of genes involved in the anthocyanin biosynthetic pathway and anthocyanin levels. However, a significant number of genes related to lignin, chlorophyll and carotenoid biosynthetic pathways were strongly correlated with the accumulation of anthocyanin.

The accumulation of anthocyanin is influenced by endogenous factors such as nitrogen, hormones, and sugars [57–60]. In our research, we identified TFs that respond to anthocyanin levels in *L. coreana*, including MYB and bHLH-type TFs, as well as C2C2 zinc finger and DELLA proteins. In *Arabidopsis*, the *AtLSD1* gene encoding a C2C2-type zinc finger protein regulates cell death and is involved in the formation of aerenchyma under waterlogged conditions [61]. In rice, the nitrogen-efficient cultivar Yangdao 6 has well-developed aerenchyma and demonstrates superior nitrate absorption and utilization compared to the nitrogen-inefficient cultivar Nongxing 57 [62]. The C2C2 zinc finger protein gene family may share a conserved function in plant-programmed cell death by influencing aerenchyma formation and nitrogen utilization. In our study, three C2C2 zinc finger proteins were identified as candidate regulators (Fig. 5).

On the other hand, DELLA proteins, a subfamily of GRAS transcription factors, integrate multiple hormone signals, including gibberellic acid (GA), auxin, and abscisic acid (ABA) [63, 64]. GA is known to negatively regulate anthocyanin synthesis [65, 66]. In our study, the expression of *GRAS-4*, a potential negative regulator of GA biosynthesis, was significantly higher in RLHT than in GLHT across all stages and positively correlated with anthocyanin content (Fig. 5C). In addition, GA-related pathways such as diterpenoid biosynthesis (ko00904) and plant hormone signal transduction (ko04075) were significantly enriched in KEGG analysis (Fig. 3D). These results suggest that endogenous GA in L. coreana may influence anthocyanin accumulation through *GRAS-4*. This inference is supported by a recent study in apple, where overexpression of *MdGA2ox7* reduced active GA levels and promoted anthocyanin accumulation [67].

ABA biosynthesis shares precursors with the carotenoid pathway, and ABA is known to induce anthocyanin synthesis [68]. This also explains the significant correlation of genes involved in carotenoid biosynthesis with anthocyanin content in *L. coreana* leaves (particularly *ABA2*). Thus, the accumulation of ABA precursors could influence the ABA and anthocyanin biosynthesis. Furthermore, the regulatory effects of ABA on anthocyanin synthesis may be enhanced by sugars, since combined ABA treatment with sugars significantly promotes the expression of many anthocyanin-related genes in *Arabidopsis* [66]. Sugars also play a critical role in anthocyanin regulation by providing carbon skeletons through the shikimate pathway and by acting as signaling molecules [69]. In our study, pathways related to sugar metabolism, such as glyoxylate and dicarboxylate metabolism (ko00630) and starch and sucrose metabolism (ko00500) were enriched (Fig. 3D), supporting the participation of endogenous sugars in the accumulation of anthocyanin in *L. coreana* leaves.

**Fig. 7:**
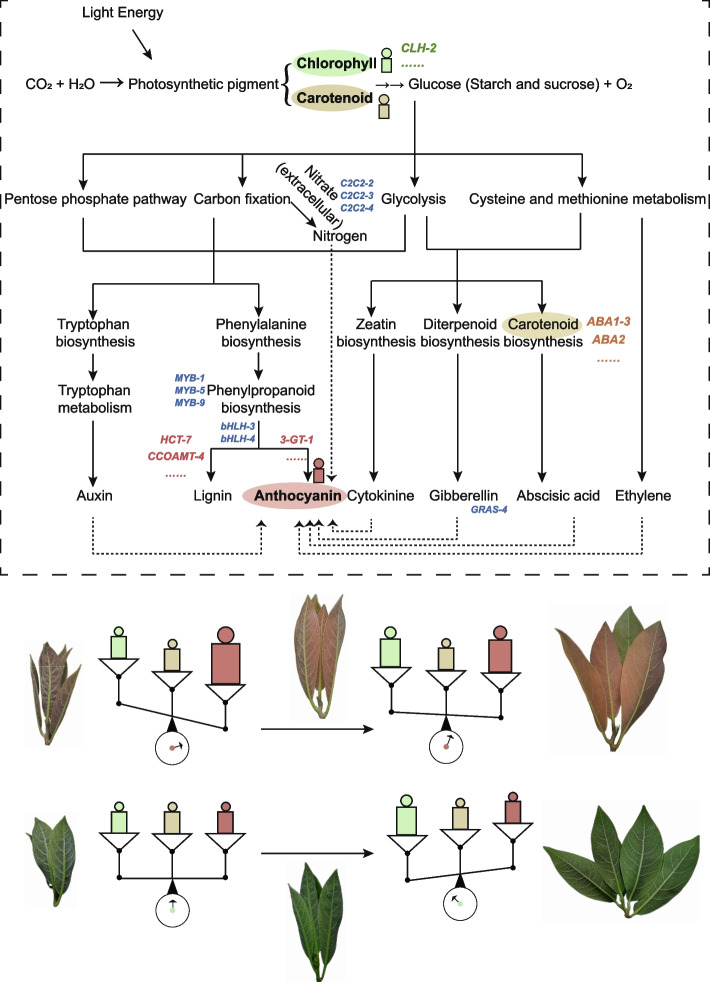
Model of the mechanism underlying leaf color change in *L. coreana*. The content within the dashed box represents hypotheses that require further validation; the dashed arrows indicate endogenous substances that may influence anthocyanin accumulation in *L. coreana*; the solid arrows depict the sequential steps of the biosynthetic pathways. The size of the weights represents the pigment content at each developmental stage

The synthesis of plant sugars is closely linked to photosynthesis, which depends on chlorophyll and carotenoids for light absorption [70]. Therefore, the regulation of anthocyanin accumulation during leaf development in L. coreana is complex and likely involves interactions among photosynthetic capacity, sugar availability, and hormone signaling. The exact mechanisms require further investigation. Based on our findings, we propose a working model for leaf color change in *L. coreana* (Fig. 7), summarizing the expression patterns of key structural genes and transcription factors across developmental stages.

## Conclusion

This study compared red-leaf (RLHT) and green-leaf (GLHT) hawk tea across three developmental stages. In RLHT, three anthocyanins (cyanidin-3-*O*-glucoside, cyanidin-3-*O*-rutinoside, and pelargonidin-3-*O*-glucoside) accumulated to high levels at the young stage and declined as leaves matured, while GLHT maintained low levels throughout. The late biosynthetic genes *ANR* and *3GT* were upregulated in RLHT and correlated with anthocyanin content, whereas lignin pathway genes (*HCT*, *CCOAMT*) were downregulated, indicating a shift in carbon flux toward anthocyanin synthesis. Nine transcription factors (three MYB, two bHLH, three C2C2 zinc finger, and one GRAS) showed expression patterns consistent with regulation of anthocyanin biosynthesis. Among them, *MYB-9*, *bHLH-4* and *GRAS-4* were highly expressed in RLHT and positively correlated with anthocyanin content, suggesting involvement in GA-mediated color regulation. Overall, these findings identify key metabolites, genes, and transcription factors that distinguish red from green hawk tea leaves, providing a basis for breeding high-anthocyanin varieties with stable leaf color traits.

## Supplementary Information

Supplementary Material 1: Fig. S1: The leaf phenotype on seedlings of *L. coreana*. Fig. S2: RNA Quality Agilent detection map. Fig. S3: Real-time qPCR melting curve of key structural genes. Fig. S4: Heatmaps of differentially expressed genes in the three pigment biosynthesis pathway. Table S1: RNA Sample Quality Control Report. Table S2: List of primers used in this study. Table S3: 42 flavonoid metabolites in the leaves of the sampled *L. coreana*. Table S4: 29 anthocyanin differential accumulated metabolites in the group. Table S5: Transcriptome Data Quality Analysis. Table S6: Data filtering statistics. Table S7: Statistical table of KEGG enrichment analysis for all DEGs. Table S8: Spearman correlation matrix between DEGs and DAMs and pigment content.

## Data Availability

The raw RNA-Seq data of Illumina sequences have been deposited in the NCBI Sequence Read Archive under accession numbers PRJNA1235469. Metabolomics datasets and other supporting data are included in the supplementary materials of this article.
