# Supplementary material for: Integrated metabolomic and transcriptomic analyses provide new perspectives into the discoloration of hawk tea tender leaves
Source: BMC Plant Biol. 2026 May 14;26:1186. doi: 10.1186/s12870-026-08888-x (PMC13359805; doi:10.1186/s12870-026-08888-x)
Supplement: Supplementary file 1 — Supplementary Material 1: Fig. S1: The leaf phenotype on seedlings of L. coreana. Fig. S2: RNA Quality Agilent detection map. Fig. S3: Real-time qPCR melting curve of key structural genes. Fig. S4: Heatmaps of differentially expressed genes in the three pigment biosynthesis pathway. Table S1: RNA Sample Quality Control Report. Table S2: List of primers used in this study. Table S3: 42 flavonoid metabolites in the leaves of the sampled L. coreana. Table S4: 29 anthocyanin differential accumulated metabolites in the group. Table S5: Transcriptome Data Quality Analysis. Table S6: Data filtering statistics. Table S7: Statistical table of KEGG enrichment analysis for all DEGs. Table S8: Spearman correlation matrix between DEGs and DAMs and pigment content. [file 12870_2026_8888_MOESM1_ESM.zip › Fig. S4.pdf]

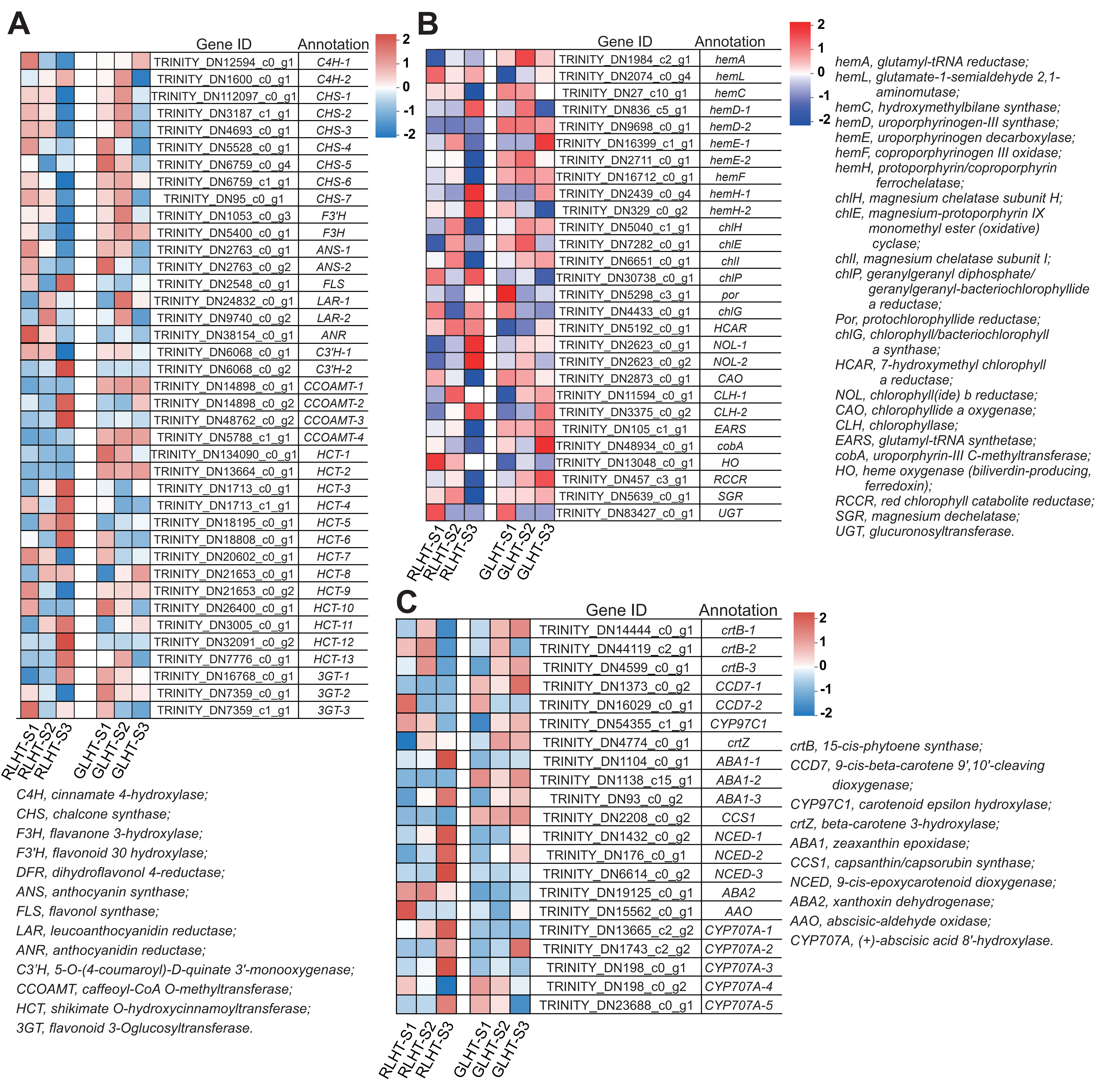
A
Gene ID
Annotation
2
1
0
-1
-2
TRINITY_DN12594_c0_g1 C4H-1
TRINITY_DN1600_c0_g1 C4H-2
TRINITY_DN112097_c0_g1 CHS-1
TRINITY_DN3187_c1_g1 CHS-2
TRINITY_DN4693_c0_g1 CHS-3
TRINITY_DN5528_c0_g1 CHS-4
TRINITY_DN6759_c0_g4 CHS-5
TRINITY_DN6759_c1_g1 CHS-6
TRINITY_DN95_c0_g1 CHS-7
TRINITY_DN1053_c0_g3 F3'H
TRINITY_DN5400_c0_g1 F3H
TRINITY_DN2763_c0_g1 ANS-1
TRINITY_DN2763_c0_g2 ANS-2
TRINITY_DN2548_c0_g1 FLS
TRINITY_DN24832_c0_g1 LAR-1
TRINITY_DN9740_c0_g2 LAR-2
TRINITY_DN38154_c0_g1 ANR
TRINITY_DN6068_c0_g1 C3'H-1
TRINITY_DN6068_c0_g2 C3'H-2
TRINITY_DN14898_c0_g1 CCOAMT-1
TRINITY_DN14898_c0_g2 CCOAMT-2
TRINITY_DN48762_c0_g2 CCOAMT-3
TRINITY_DN5788_c1_g1 CCOAMT-4
TRINITY_DN134090_c0_g1 HCT-1
TRINITY_DN13664_c0_g1 HCT-2
TRINITY_DN1713_c0_g1 HCT-3
TRINITY_DN1713_c1_g1 HCT-4
TRINITY_DN18195_c0_g1 HCT-5
TRINITY_DN18808_c0_g1 HCT-6
TRINITY_DN20602_c0_g1 HCT-7
TRINITY_DN21653_c0_g1 HCT-8
TRINITY_DN21653_c0_g2 HCT-9
TRINITY_DN26400_c0_g1 HCT-10
TRINITY_DN3005_c0_g1 HCT-11
TRINITY_DN32091_c0_g2 HCT-12
TRINITY_DN7776_c0_g1 HCT-13
TRINITY_DN16768_c0_g1 3GT-1
TRINITY_DN7359_c0_g1 3GT-2
TRINITY_DN7359_c1_g1 3GT-3
RLHT-S1
RLHT-S2
RLHT-S3
GLHT-S1
GLHT-S2
GLHT-S3
C4H, cinnamate 4-hydroxylase;
CHS, chalcone synthase;
F3H, flavanone 3-hydroxylase;
F3'H, flavonoid 30 hydroxylase;
DFR, dihydroflavonol 4-reductase;
ANS, anthocyanin synthase;
FLS, flavonol synthase;
LAR, leucoanthocyanidin reductase;
ANR, anthocyanidin reductase;
C3'H, 5-O-(4-coumaroyl)-D-quinate 3'-monooxygenase;
CCOAMT, caffeoyl-CoA O-methyltransferase;
HCT, shikimate O-hydroxycinnamoyltransferase;
3GT, flavonoid 3-Oglucosyltransferase.
B
Gene ID
Annotation
2
1
0
-1
-2
TRINITY_DN1984_c2_g1 hemA
TRINITY_DN2074_c0_g4 hemL
TRINITY_DN27_c10_g1 hemC
TRINITY_DN836_c5_g1 hemD-1
TRINITY_DN9698_c0_g1 hemD-2
TRINITY_DN16399_c1_g1 hemE-1
TRINITY_DN2711_c0_g1 hemE-2
TRINITY_DN16712_c0_g1 hemF
TRINITY_DN2439_c0_g4 hemH-1
TRINITY_DN329_c0_g2 hemH-2
TRINITY_DN5040_c1_g1 chlH
TRINITY_DN7282_c0_g1 chlE
TRINITY_DN6651_c0_g1 chlI
TRINITY_DN30738_c0_g1 chlP
TRINITY_DN5298_c3_g1 por
TRINITY_DN4433_c0_g1 chlG
TRINITY_DN5192_c0_g1 HCAR
TRINITY_DN2623_c0_g1 NOL-1
TRINITY_DN2623_c0_g2 NOL-2
TRINITY_DN2873_c0_g1 CAO
TRINITY_DN11594_c0_g1 CLH-1
TRINITY_DN3375_c0_g2 CLH-2
TRINITY_DN105_c1_g1 EARS
TRINITY_DN48934_c0_g1 cobA
TRINITY_DN13048_c0_g1 HO
TRINITY_DN457_c3_g1 RCCR
TRINITY_DN5639_c0_g1 SGR
TRINITY_DN83427_c0_g1 UGT
RLHT-S1
RLHT-S2
RLHT-S3
GLHT-S1
GLHT-S2
GLHT-S3
hemA, glutamyl-tRNA reductase;
hemL, glutamate-1-semialdehyde 2,1-aminomutase;
hemC, hydroxymethylbilane synthase;
hemD, uroporphyrinogen-III synthase;
hemE, uroporphyrinogen decarboxylase;
hemF, coproporphyrinogen III oxidase;
hemH, protoporphyrin/coproporphyrin ferrochelatase;
chlH, magnesium chelatase subunit H;
chlE, magnesium-protoporphyrin IX monomethyl ester (oxidative) cyclase;
chlI, magnesium chelatase subunit I;
chlP, geranylgeranyl diphosphate/ geranylgeranyl-bacteriochlorophyllide a reductase;
Por, protochlorophyllide reductase;
chlG, chlorophyll/bacteriochlorophyll a synthase;
HCAR, 7-hydroxymethyl chlorophyll a reductase;
NOL, chlorophyll(ide) b reductase;
CAO, chlorophyllide a oxygenase;
CLH, chlorophyllase;
EARS, glutamyl-tRNA synthetase;
cobA, uroporphyrin-III C-methyltransferase;
HO, heme oxygenase (biliverdin-producing, ferredoxin);
RCCR, red chlorophyll catabolite reductase;
SGR, magnesium dechelatase;
UGT, glucuronosyltransferase.
C
Gene ID
Annotation
2
1
0
-1
-2
TRINITY_DN14444_c0_g1 crtB-1
TRINITY_DN44119_c2_g1 crtB-2
TRINITY_DN4599_c0_g1 crtB-3
TRINITY_DN1373_c0_g2 CCD7-1
TRINITY_DN16029_c0_g1 CCD7-2
TRINITY_DN54355_c1_g1 CYP97C1
TRINITY_DN4774_c0_g1 crtZ
TRINITY_DN1104_c0_g1 ABA1-1
TRINITY_DN1138_c15_g1 ABA1-2
TRINITY_DN93_c0_g2 ABA1-3
TRINITY_DN2208_c0_g2 CCS1
TRINITY_DN1432_c0_g2 NCED-1
TRINITY_DN176_c0_g1 NCED-2
TRINITY_DN6614_c0_g2 NCED-3
TRINITY_DN19125_c0_g1 ABA2
TRINITY_DN15562_c0_g1 AAO
TRINITY_DN13665_c2_g2 CYP707A-1
TRINITY_DN1743_c2_g2 CYP707A-2
TRINITY_DN198_c0_g1 CYP707A-3
TRINITY_DN198_c0_g2 CYP707A-4
TRINITY_DN23688_c0_g1 CYP707A-5
RLHT-S1
RLHT-S2
RLHT-S3
GLHT-S1
GLHT-S2
GLHT-S3
crtB, 15-cis-phytoene synthase;
CCD7, 9-cis-beta-carotene 9',10'-cleaving dioxygenase;
CYP97C1, carotenoid epsilon hydroxylase;
crtZ, beta-carotene 3-hydroxylase;
ABA1, zeaxanthin epoxidase;
CCS1, capsanthin/capsorubin synthase;
NCED, 9-cis-epoxycarotenoid dioxygenase;
ABA2, xanthoxin dehydrogenase;
AAO, abscisic-aldehyde oxidase;
CYP707A, (+)-abscisic acid 8'-hydroxylase.
