# Supplementary figures and images for: Integrated metabolomic and transcriptomic analyses provide new perspectives into the discoloration of hawk tea tender leaves
Source: BMC Plant Biol. 2026 May 14;26:1186. doi: 10.1186/s12870-026-08888-x (PMC13359805; doi:10.1186/s12870-026-08888-x)

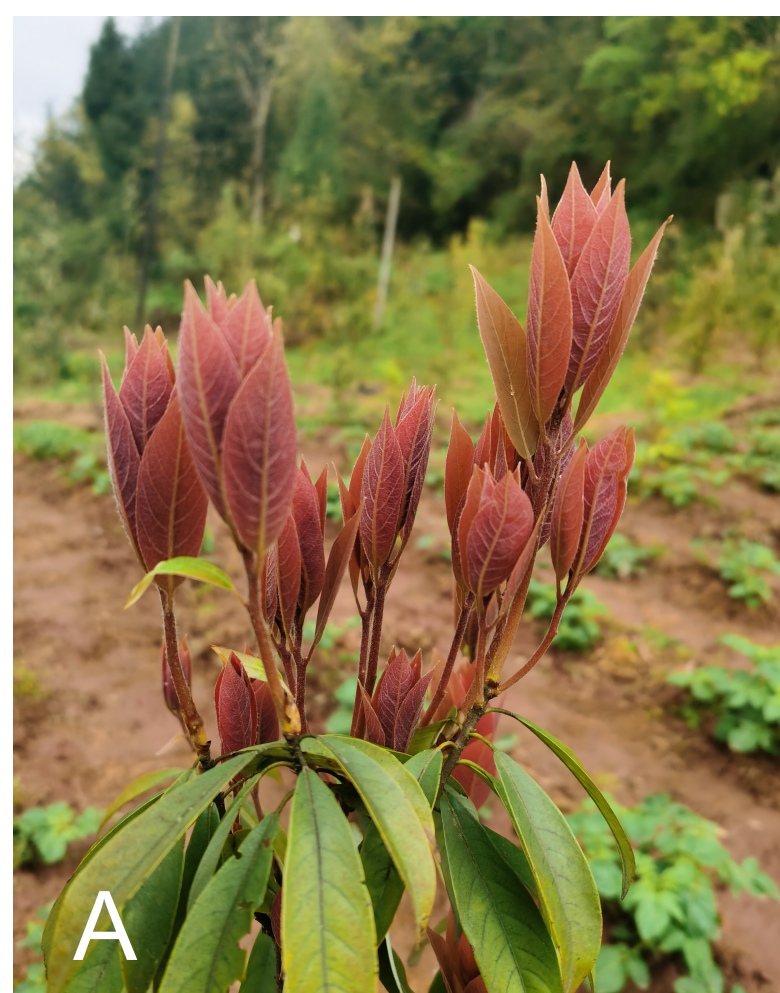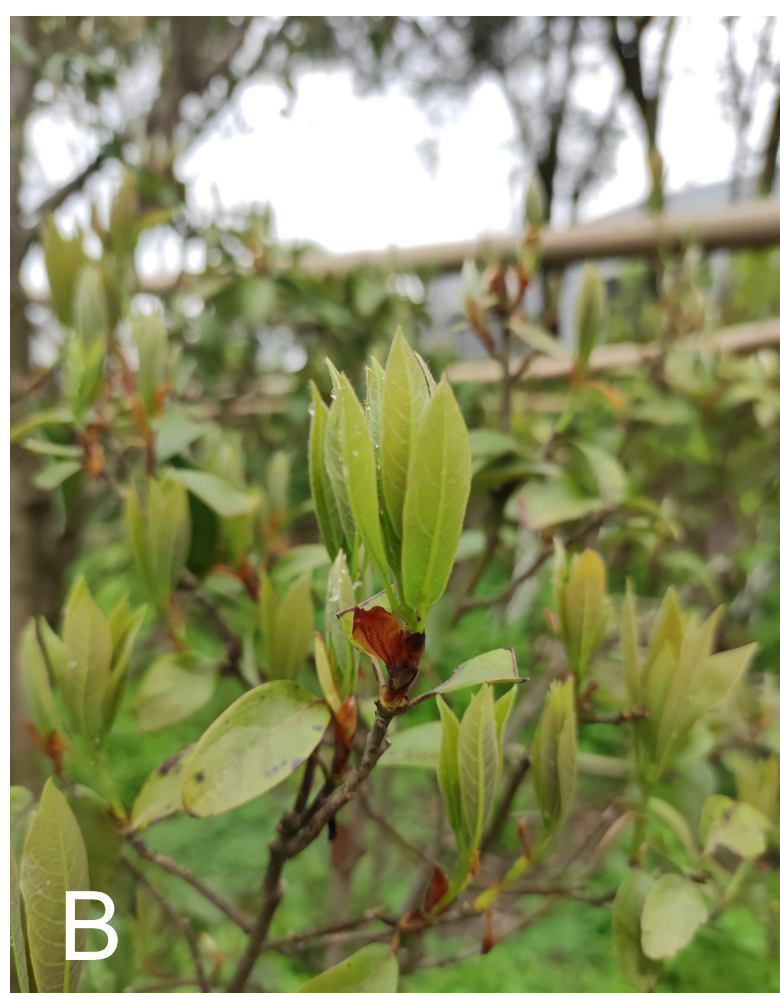

**Fig. S1.** The leaf phenotype on seedlings of *L. coreana*.

Supplement: Supplementary file 1 — Supplementary Material 1: Fig. S1: The leaf phenotype on seedlings of L. coreana. Fig. S2: RNA Quality Agilent detection map. Fig. S3: Real-time qPCR melting curve of key structural genes. Fig. S4: Heatmaps of differentially expressed genes in the three pigment biosynthesis pathway. Table S1: RNA Sample Quality Control Report. Table S2: List of primers used in this study. Table S3: 42 flavonoid metabolites in the leaves of the sampled L. coreana. Table S4: 29 anthocyanin differential accumulated metabolites in the group. Table S5: Transcriptome Data Quality Analysis. Table S6: Data filtering statistics. Table S7: Statistical table of KEGG enrichment analysis for all DEGs. Table S8: Spearman correlation matrix between DEGs and DAMs and pigment content. [file 12870_2026_8888_MOESM1_ESM.zip › Fig. S1.pdf]

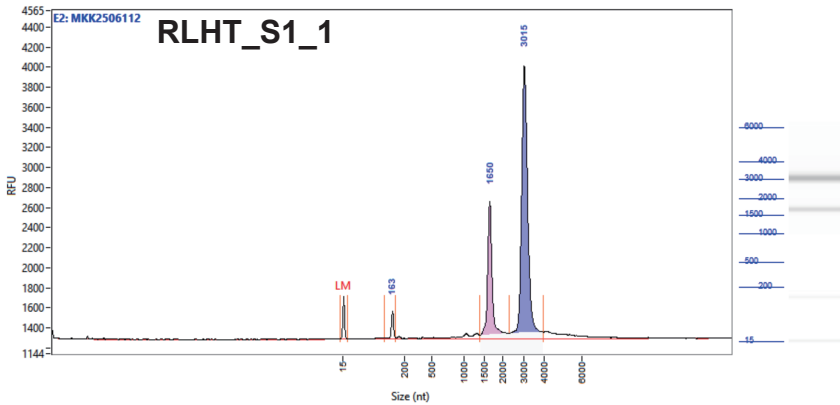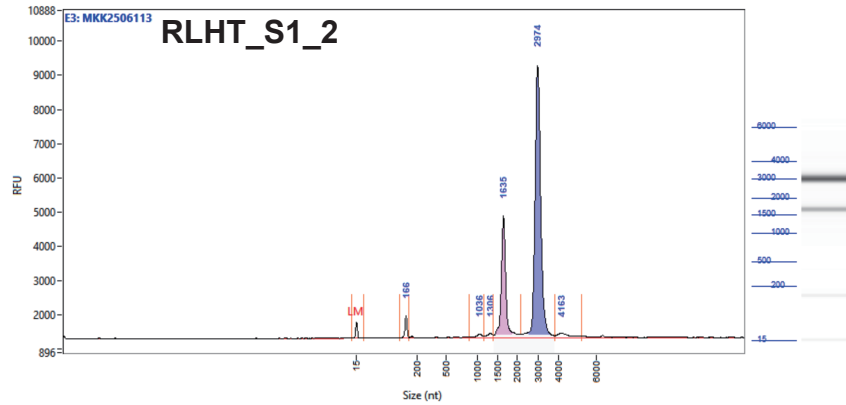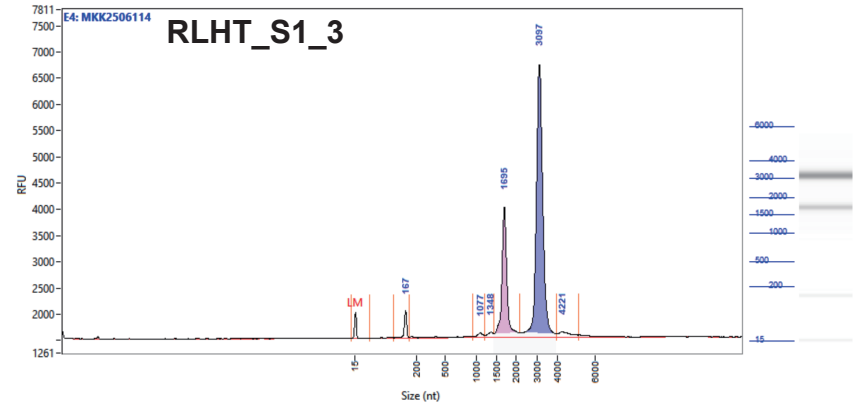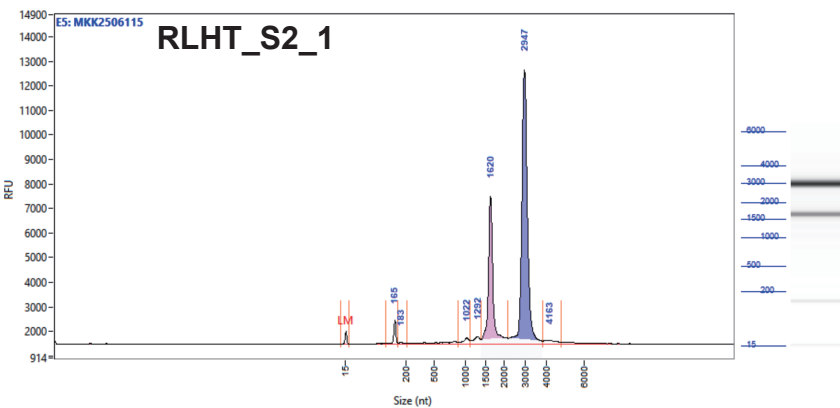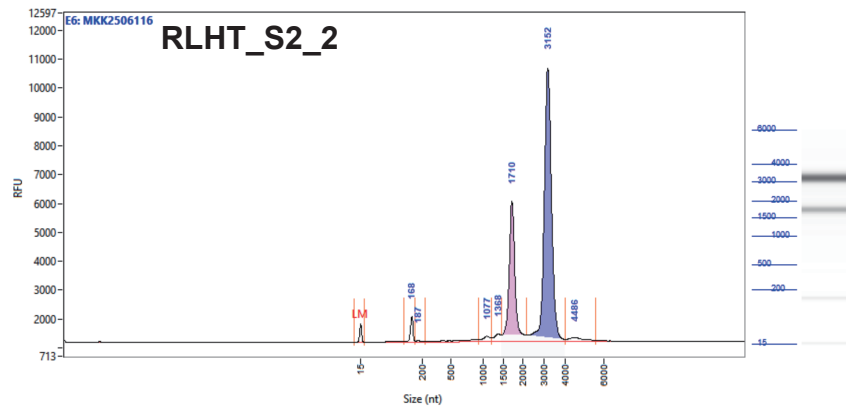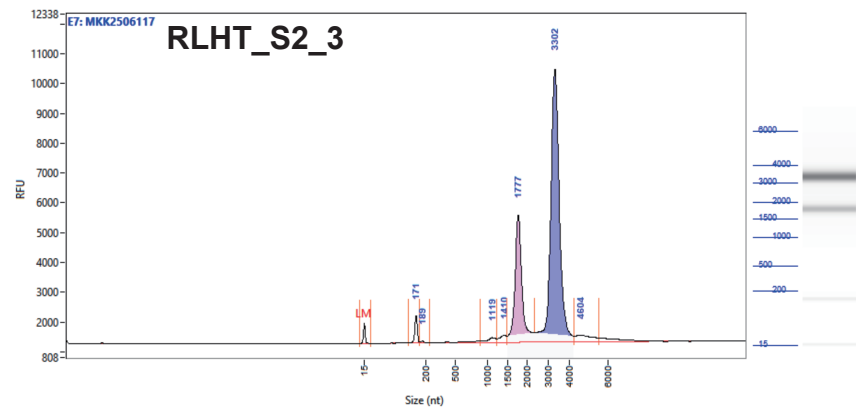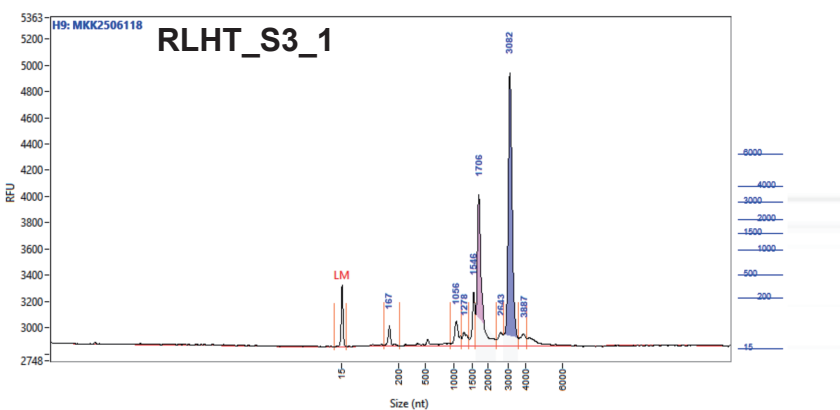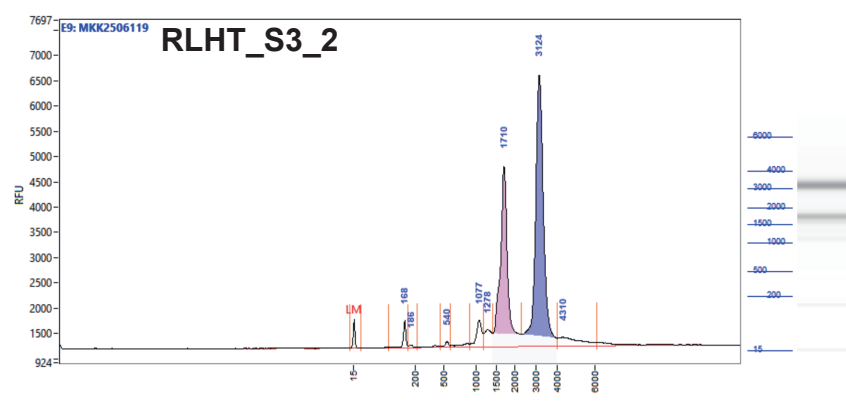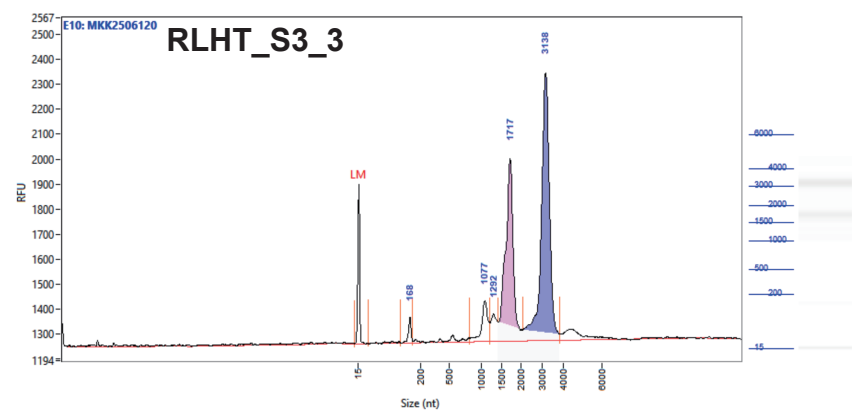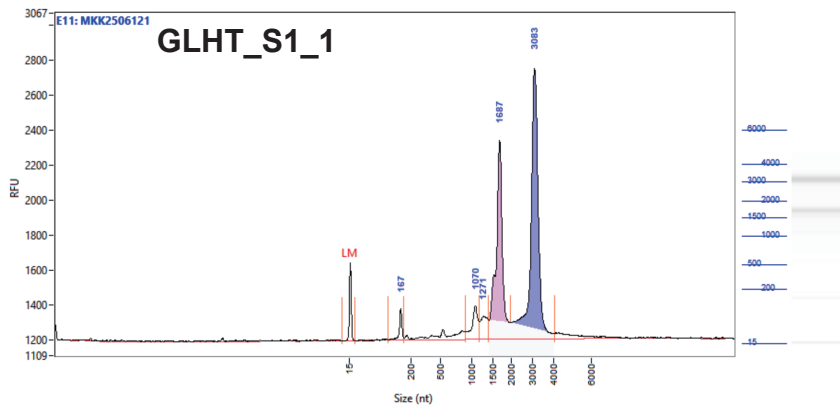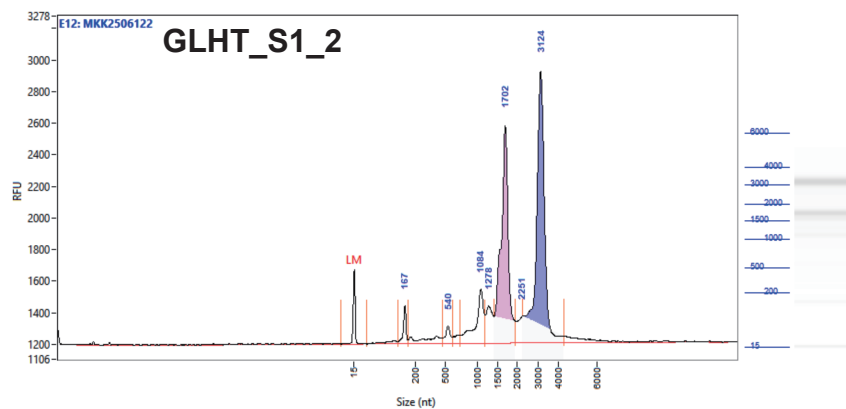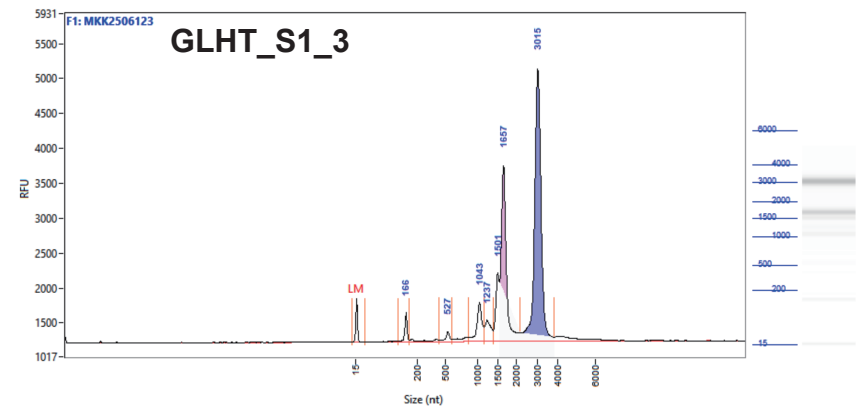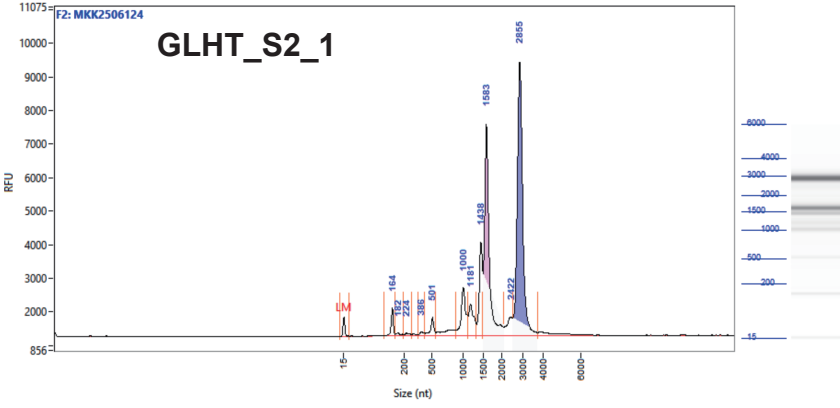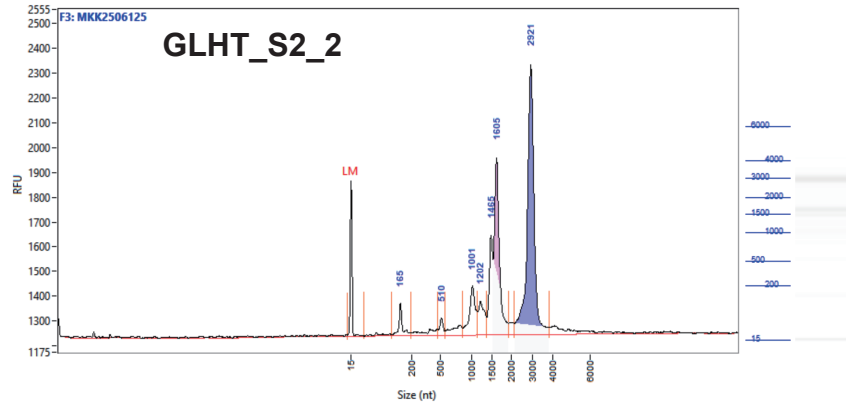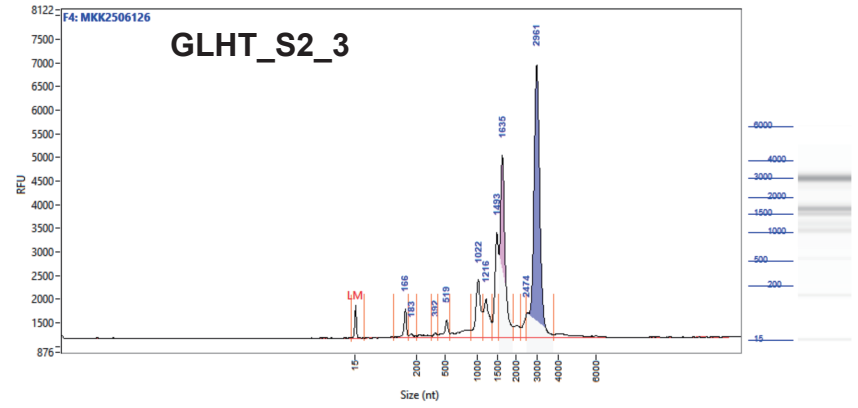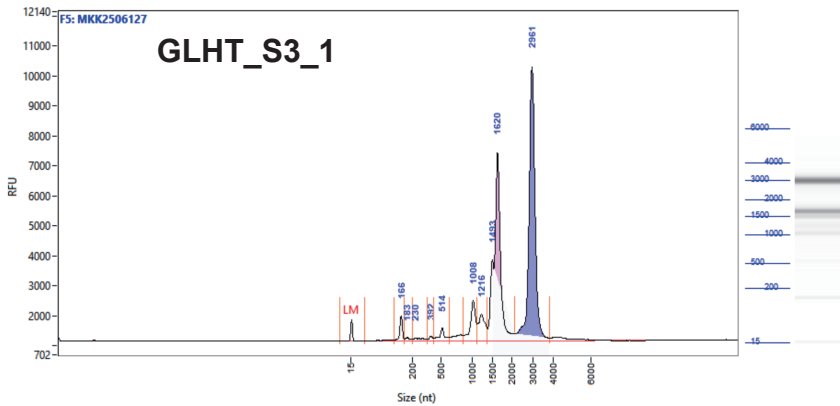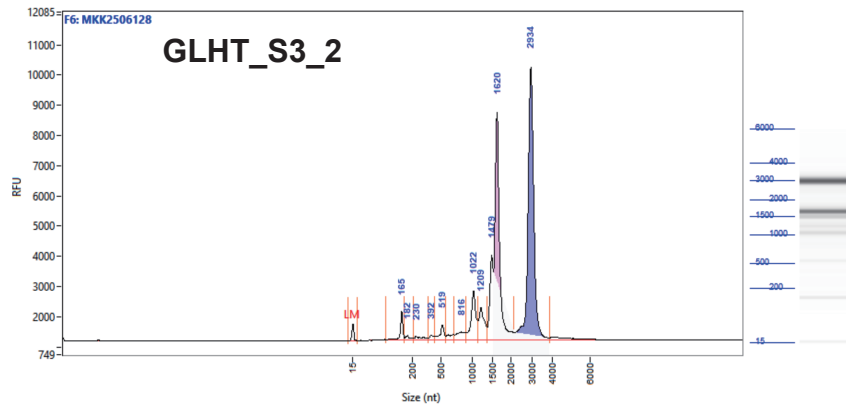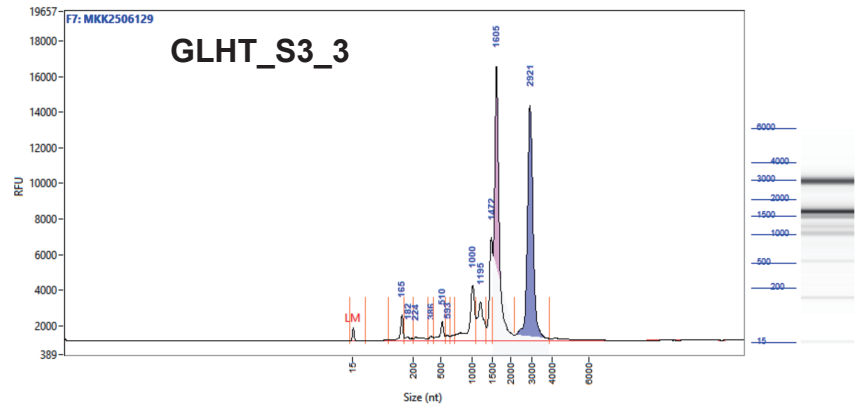

Supplement: Supplementary file 1 — Supplementary Material 1: Fig. S1: The leaf phenotype on seedlings of L. coreana. Fig. S2: RNA Quality Agilent detection map. Fig. S3: Real-time qPCR melting curve of key structural genes. Fig. S4: Heatmaps of differentially expressed genes in the three pigment biosynthesis pathway. Table S1: RNA Sample Quality Control Report. Table S2: List of primers used in this study. Table S3: 42 flavonoid metabolites in the leaves of the sampled L. coreana. Table S4: 29 anthocyanin differential accumulated metabolites in the group. Table S5: Transcriptome Data Quality Analysis. Table S6: Data filtering statistics. Table S7: Statistical table of KEGG enrichment analysis for all DEGs. Table S8: Spearman correlation matrix between DEGs and DAMs and pigment content. [file 12870_2026_8888_MOESM1_ESM.zip › Fig. S2-rna quality-20251213.pdf]
